# Prognostic Value and Immunological Role of MORF4-Related Gene-Binding Protein in Human Cancers

**DOI:** 10.3389/fcell.2021.703415

**Published:** 2021-09-29

**Authors:** Dongqi Chai, Lilong Zhang, Yongjun Guan, Jingping Yuan, Man Li, Weixing Wang

**Affiliations:** ^1^Department of Hepatobiliary and Laparoscopic Surgery, Renmin Hospital of Wuhan University, Wuhan, China; ^2^Department of Pathology, Renmin Hospital of Wuhan University, Wuhan, China

**Keywords:** MRGBP, cancer, prognosis, immune infiltration, macrophage

## Abstract

MORF4-related gene-binding protein (MRGBP) is the subunit of the NuA4 histone acetyltransferase complex which is involved in transcriptional activation of select genes principally by acetylation of nucleosomal histones H4 and H2A. Much of the research indicated an oncogenic role of MRGBP in the development of cancers. However, it is still unknown the role MRGBP plays in human cancers, which deserves further exploration. In this research, the expression profile, prognostic value of MRGBP, and the relationship between MRGBP and immune infiltration were explored in 33 types of cancer. The differences in MRGBP expression in tumor and normal tissues were explored using data from The Cancer Genome Atlas, Gene Expression Omnibus and ONCOMINE. Analysis of the association between MRGBP and prognosis using Kaplan-Meier survival curve and COX analysis. The data of Tumor mutational burden (TMB), microsatellite instability (MSI) from TCGA. The relationship Between MRGBP expression and immunity was analyzed using the ESTIMATE algorithm and CIBERSORT. Furthermore, we explored MRGBP expression and the relationship between MRGBP expression and macrophage infiltration using immunohistochemical analysis in lower grade glioma (LGG). Our results revealed that MRGBP was highly expressed in most cancer tissues compared with normal tissues. Tumors with increased MRGBP expression had a high clinicopathologic stage and poor prognosis. The expression of MRGBP was closely related to the TMB, MSI. We also found a significant negative correlation between MRGBP expression and stromal scores and immune scores in various types of cancer. Furthermore, MRGBP expression was associated with a variety of immune cells including B cells, NK cells, T cells, and macrophages. LGG and LIHC was selected as representative cancer types for further study, the results of immunohistochemistry indicated that the protein levels of MRGBP were significantly elevated in tumor tissues. Moreover, our LIHC data analysis showed that patients with high MRGBP expression were associated with short survival rates and MRGBP was a risk factor to determine OS. Immunohistochemistry also confirmed that M0 macrophage infiltration in the MRGBP-high group significantly increased. In conclusion, these results reveal that MRGBP can serve as a potential prognostic biomarker and it plays an important role in tumor immune infiltration in various tumors, especially in LGG and LIHC.

## Introduction

MORF4-related gene-binding protein (MRGBP), also known as chromosome 20 open reading frame 20 (C20orf20), encodes a subunit of the TRRAP/TIP60 histone acetyltransferase complex and binds directly to MRG15 and MRGX proteins (Cai et al., 2003). The level of MRGBP expression was relatively low in normal tissues except testis. However, in most colorectal cancers, MRGBP expression is increased and it promotes colorectal carcinogenesis by providing an advantage in cell proliferation and cancer cell division (Carvalho et al., 2009; Yamaguchi et al., 2010, 2011). Furthermore, patients with higher TNM stage and T classification exhibit higher levels of MRGBP expression (Ding et al., 2017). It has also been shown that MRGBP accelerates androgen receptor-mediated transactivation and promotes the growth of androgen receptor-positive prostate cancer cells (Ito et al., 2018). Another tumor, cutaneous squamous cell carcinoma, in which MRGBP is thought to be overexpressed. When MRGBP is inhibited it induces apoptosis and reduces tumor growth (Watt et al., 2011). Also, it is associated with the progression of lung and cervical cancers (Scotto et al., 2008; Dai et al., 2019). MRGBP is involved in a variety of tumor processes, and its expression is significantly increased in a variety of tumors, suggesting that MRGBP may be used as a diagnostic biomarker and therapeutic target for tumors. However, the role of MRGBP in tumors has only been studied in a few tumors, and the link between MRGBP and various cancers deserves to be investigated.

Immune checkpoint proteins generate co-stimulatory or inhibitory signals in the immune response and modulate the host immune response under normal conditions. Recent studies have focused on the immune checkpoint PD-1 and its ligand PD-L1 signaling axis, which are highly expressed in tumors and can bind to PD-1 on the surface of T cells, limiting T cell activation and inducing a depleted state leading to tumor immune escape, Activation of T cell immune response by anti-CTLA-4 monoclonal antibody clears Treg cells (Lingel and Brunner-Weinzierl, 2019; Xin Yu et al., 2020). Recent studies have found that immunotherapy that blocks these receptors has shown good efficacy in treating several cancers such as melanoma, non-small cell lung cancer, kidney cancer, and bladder cancer (Topalian et al., 2012; Luke et al., 2017; Rizvi et al., 2018). The combination of targeted blockade of PD-1 and CTLA-4 is also a synergistic treatment option with good efficacy. The tumor microenvironment (TME) is composed of tumor cells, stromal cells, immune cells and their secreted factors. Immune cells play a dual role in inhibiting tumor progression and assisting tumor immune evasion (Junttila and de Sauvage, 2013). Immunotherapy for tumors is to target immunosuppressive cells in the immune microenvironment. However, there are a significant number of patients remain unresponsive to currently available immunotherapies which may be caused by the heterogeneity of the immune microenvironment.

In this study, our data were derived from a combination of public databases and web tools, and R language was used to analyze the association between MRGBP expression and patient prognosis and its potential role in tumor immunity.

## Materials and Methods

### Expression Analysis of MORF4-Related Gene-Binding Protein

Data from the Genotype-Tissue Expression (GTEx) portal and the Cancer Cell Line Encyclopedia (CCLE) database were used to analyze MRGBP gene expression in normal tissues and tumor cell lines. Differential MRGBP expression between tumor and adjacent normal tissue was assessed by setting the threshold fold change to 1.5 and placing a *p*-value cutoff of 0.05 in the ONCOMINE database. The UCSC Xena was chosen to obtained RNA sequences associated with 33 cancers. We extracted and integrated MRGBP expression levels by Perl software for analysis of pan-cancer. The Wilcox. test method was used to analyze the differences in mRNA expression in different cancer types. The box plot is generated with the R-package “gg pubr.” We combined the GTEx database and The Cancer Genome Atlas (TCGA) database to further explore the differences between cancer and normal tissue. Validation of differential expression of MRGBP between normal and tumor tissues in the GEO database using GSE16011. The box plot is implemented by the R software package “ggplot2.”

### Correlation of the MORF4-Related Gene-Binding Protein Expression to Clinical Phenotype and Prognosis

Next, we explored the association between MRGBP expression and clinical outcomes through survival information obtained from the TCGA database. The relationship between mRNA expression levels and patient survival rate was investigated by overall survival (OS), disease-specific survival (DSS), disease-free interval (DFI), and progression-free interval (PFI). The relationship between MRGBP expression and survival outcome in different cancer types we performed by Kaplan-Meier, log-rank test and COX analysis, KM curves, and forest plots were plotted using the R program (v4.0.3). Subsequently, we used “limma” and “ggpubr” in R to perform a correlation analysis of clinicopathological characteristics. *p* < 0.05 were considered significant. The correlation of MRGBP levels to patients’ OS and PFS was further analyzed using Kaplan-Meier Plotter, which has data from 3 databases including Gene Expression Omnibus (GEO), European Genome-phenome Archive (EGA), and TCGA. Validation of the prognostic value of MRGBP in LGG using the Chinese Glioma Genome Altas (CGGA) and GEO dataset (GSE 4412). The survival curves were implemented by the R software package “survival” and “survminer.”

### Mutation Analysis of MORF4-Related Gene-Binding Protein

We perform mutation analysis of MRGBP in the CBio Cancer Genome Portal which is an open platform that provides visualization and analysis of gene copy number alterations and mutations in various cancers (Cerami et al., 2012). Catalog of Somatic Mutations in Cancer is a detailed and comprehensive resource for exploring the effect of somatic mutations in human cancer (Tate et al., 2019). Therefore, we investigated MRGBP mutations in various cancer types using COSMIC.

### Relationship Between MORF4-Related Gene-Binding Protein Expression and Tumor Mutation Burden Tumor Microsatellite Instability and Some Specific Genes

We calculate the number of mutations in 33 types of cancer by counting the TMB and dividing it by the total length of the exons according to the Perl script. We used the somatic mutation data obtained from TCGA to identify the MSI scores for each tumor sample. We performed correlation analysis between cancer gene expression and TMB or MSI using the Spearman method, and the results were visualized by the “fmsb” in the R program.

Data for 5 MMR genes (MLH1, MSH2, MSH6, PMS2, and EPCAM), 5 methyltransferases genes (DNMT1, TRDMT1, DNMT3A, DNMT3B, DNMT3L), and 13 m6A-related genes were all downloaded from TCGA. We used Pearson correlation analysis to assess the relationship between MRGBP expression and the level of mutations in the genes mentioned above.

### Relationship Between MORF4-Related Gene-Binding Protein Expression and Immunity

The percentage of stromal and immune cells in TME is expressed by the mesenchymal score and the immune score. ESTIMATE score uses available gene expression profiles to calculate the extent of mesenchymal or immune cell infiltration in the tumor, which indirectly represents tumor purity. In this research, the relationship between MRGBP levels and these two scores were calculated using the ESTIMATE algorithm in the R program we downloaded. CIBERSORT is a tool that can be used to calculate the relative fraction of specific immune cells based on gene expression data. Hence, we use it to evaluate the immune cell infiltration in each tumor. Moreover, the relationship between MRGBP and immune-related genes was analyzed using the “limma” and visualized using the “reshape2” and “RColorBreyer” in R software.

### Enrichment Analysis of MORF4-Related Gene-Binding Protein-Related Gene

We acquired MRGBP binding proteins and constructed a PPI network using the STRING website (Szklarczyk et al., 2015). GEPIA2 was used to obtain the top 5 target genes associated with MRGBP and Pearson correlation analysis between MRGBP and target genes was then performed using the “Correlation Analysis” module in GEPIA2. Besides, the heatmap which showed the correlation between the top 5 target gene and 33 cancer types were obtained from TIMER2. Gene ontology (GO) and Kyoto Encyclopedia of Genes and Genomes (KEGG) gene sets were downloaded from the Gene Set Enrichment Analysis (GSEA) website. Both KEGG and GO enrichment pathway as well as functional annotation analysis of MRGBP were conducted using “clusterProfiler,” “org.Hs.eg.db,” “enrichplot,” “DOSE,” “colorspace,” “stringi,” and “ggplot2” R software package. The functional status of MRGBP in multiple cancer types was estimated by CancerSEA (filtered by correlation strength > 0.3 and false discovery rate < 0.05).

### Immunohistochemical Analysis

We collected tumors and paracancerous tissues from LGG, COAD, and READ at the People’s Hospital of Wuhan University, China, and 20 pairs of each tumor type were collected to verify the differential expression of MRGBP. In addition, we collected 10 tumor tissue specimens from LGG, totaling 30 samples, to verify the relationship between MRGBP expression and macrophage infiltration. The LIHC tumor tissues and adjacent normal tissues of 125 patients were collected from resected specimens at Tongji Hospital of Huazhong University of Science and Technology between 2012 and 2016. Tissue microarray plates containing 125 LIHC cases were constructed from paraffin-embedded LIHC tissues. Among them, 20 pairs of tissues without normal structure were excluded, and a total of 105 cases of LIHC were included. The association between MRGBP expression and macrophage infiltration in LIHC was validated using 8 MRGBP high and 8 low expressing tumor tissue samples, which were selected based on the results of tissue microarrays. For IHC, following deparaffinization, hydration, and epitope retrieval, the activity of endogenous peroxidase in the slices was inhibited for 15 min by 3% hydrogen peroxide. Then, slides were incubated overnight at 4°C with the primary antibody MRGBP (1:200, Santacruz, sc-32757) and CD68 (1:200, Abcam, ab955) in a humidity box, and subsequently placed in secondary antibody. Finally, the slides were visualized by diaminobenzidine and counterstained with hematoxylin. Immunohistochemical sections were observed by an Olympus BX63 microscope and the quantitative analysis of slides also used Image J software. Data were expressed as the mean ± standard deviation. Statistical measurements were performed using the SPSS 21.0 statistical software (SPSS Inc., Chicago, United States).

See Supplementary Table 1 for details of the database sites used in this study. Table 1 provided the full cancer type name corresponding to each abbreviation listed in the legend and the text. *P* < 0.05 in this article was considered statistically significant.

**TABLE 1:** Pan-cancer data primary from TCGA database.

Abbreviation	Full name
ACC	Adrenocortical carcinoma
BLCA	Bladder urothelial carcinoma
BRCA	Breast invasive carcinoma
CESC	Cervical squamous cell carcinoma and endocervical adenocarcinoma
CHOL	Cholangiocarcinoma
COAD	Colon adenocarcinoma
DLBC	Lymphoid neoplasm diffuse large B-cell lymphoma
ESCA	Esophageal carcinoma
GBM	Glioblastoma multiforme
HNSC	Head and neck squamous cell carcinoma
KICH	Kidney chromophobe
KIRC	Kidney renal clear cell carcinoma
KIRP	Kidney renal papillary cell carcinoma
LAML	Acute myeloid leukemia
LGG	Brain lower grade glioma
LIHC	Liver hepatocellular carcinoma
LUAD	Lung adenocarcinoma
LUSC	Lung squamous cell carcinoma
MESO	Mesothelioma
OV	Ovarian serous cystadenocarcinoma
PAAD	Pancreatic adenocarcinoma
PCPG	Pheochromocytoma and paraganglioma
PRAD	Prostate adenocarcinoma
READ	Rectum adenocarcinoma
SARC	Sarcoma
SKCM	Skin cutaneous melanoma
STAD	Stomach adenocarcinoma
TGCT	Testicular germ cell tumors
THCA	Thyroid carcinoma
THYM	Thymoma
UCEC	Uterine corpus endometrial carcinoma
UCS	Uterine carcinosarcoma
UVM	Uveal melanoma

## Results

### The Differential Expression Level of MORF4-Related Gene-Binding Protein in Human Cancers

We studied 31 types of normal tissues from GTEx databases to identify MRGBP mRNA levels. Our results indicated that MRGBP expression was commonly elevated in the bone marrow, ovary, spleen and testis (Supplementary Figure 1A). Subsequently, we investigated MRGBP expression in 39 kinds of cancer cell lines. According to CCLE analysis results, MRGBP showed significantly different expression levels among these cell lines (Supplementary Figure 1B).

**FIGURE 1:**
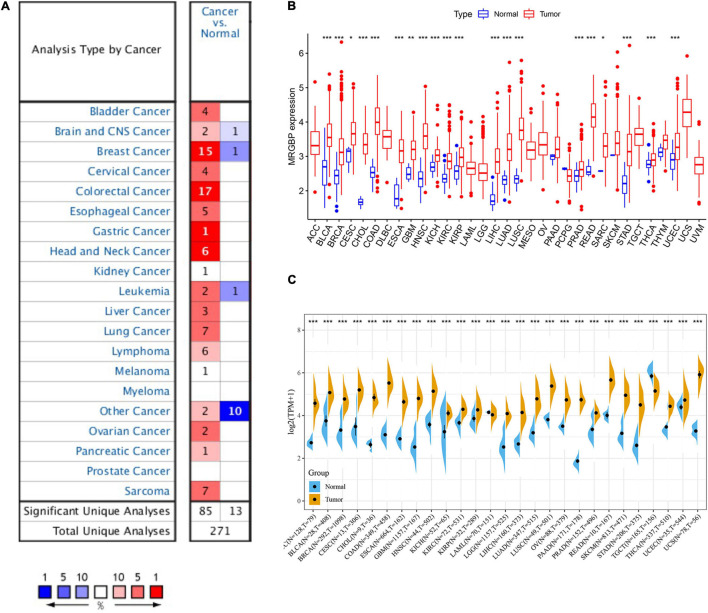
mRNA expression of MRGBP gene in various cancers and its corresponding normal tissues. **(A)** Oncomine dataset. **(B)** TCGA dataset. **(C)** TCGA and GTEx dataset (***P < 0.001, **P < 0.01, *P < 0.05).

We next evaluated the differences in MRGBP expression between the tumor and normal tissues. First, we used the Oncomine database and found that it was highly expressed in most tumors compared to normal tissues (Figure 1A). However, MRGBP was less expressed in some tumors such as brain and central nervous system cancer, breast cancer, leukemia, and other cancer. Subsequently, we used the TCGA database to further verify. Collectively, MRGBP was significantly increased in most cancers including BLCA, BRCA, CECS, CHOL, COAD, ESCA, GBM, HNSC, KICH, KIRC, KIRP, LIHC, LUAD, LUSC, PRAD, READ, SARC, STAD, THCA, and UCEC compared with paired adjacent normal tissues (Figure 1B). These results indicated that MRGBP was commonly upregulated in most tumors and was statistically significant.

We combined data from GTEx and TCGA to analyze the differential expression of MRGBP in 27 normal and tumor tissues due to the small number of normal samples in the TCGA database. The results showed that MRGBP level in tumor tissues was significantly higher than normal tissues in most tumors, which was consistent with previous results. Notably, MRGBP expression was also shown to be increased in tumors such as ACC and LGG when adding normal tissue samples (Figure 1C).

### Multifaceted Prognostic Value of MORF4-Related Gene-Binding Protein in Cancers

**FIGURE 2:**
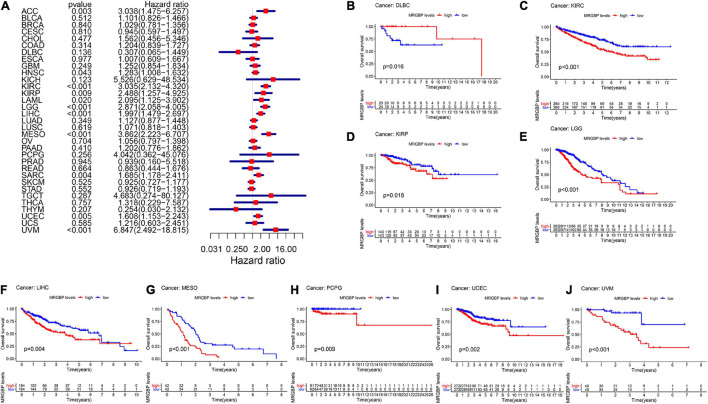
Relationship between MRGBP expression and overall survival (OS) in 33 tumors. The forest plots were calculated using univariate Cox regression **(A)** and the survival curves were calculated using Kaplan-Meier survival methods **(B–J)**.

**FIGURE 3:**
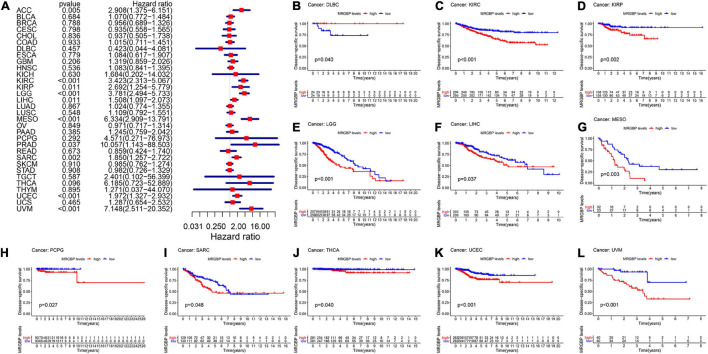
Relationship between MRGBP expression and disease-specific survival (DSS) in 33 tumors. The forest plots were calculated using univariate Cox regression **(A)** and the survival curves were calculated using Kaplan-Meier survival methods **(B–L)**.

**FIGURE 4:**
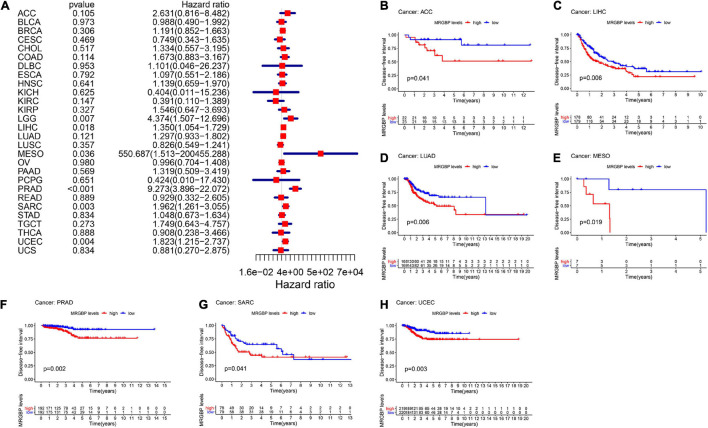
Relationship between MRGBP expression and disease-free interval (DFI) in 33 tumors. The forest plots were calculated using univariate Cox regression **(A)** and the survival curves were calculated using Kaplan-Meier survival methods **(B–H)**.

**FIGURE 5:**
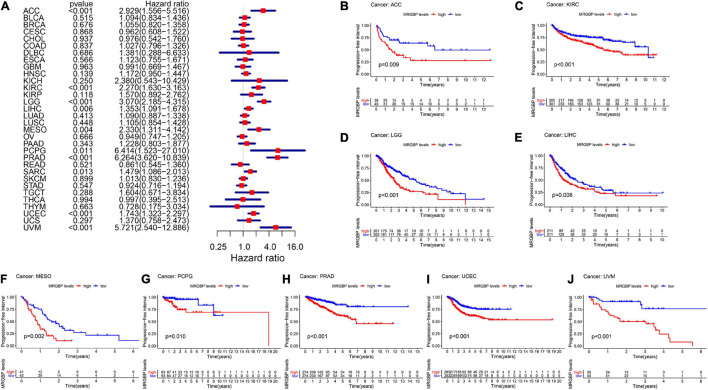
Relationship between MRGBP expression and progression-free interval (PFI) in 33 tumors. The forest plots were calculated using univariate Cox regression **(A)** and the survival curves were calculated using Kaplan-Meier survival methods **(B–J)**.

Subsequently, we analyzed the correlation between the level of MRGBP expression and prognostic value in pan-cancer by using TCGA databases. Cox regression analysis of MRGBP-related survival (OS, DSS, DFI, and PFI) indicated that high mRNA expression was a detrimental prognostic factor in ACC (OS: *p* = 0.003; DSS: *p* = 0.005; PFI: *p* < 0.001), HNSC (OS: *p* = 0.043) KIRC (OS: *p* < 0.001; DSS: *p* < 0.001; PFI: *p* < 0.001), KIRP (OS: *p* = 0.009; DSS: *p* = 0.011), LAML (OS: *p* = 0.02)LGG (OS: *p* < 0.001; DSS: *p* < 0.001; DFI: *p* = 0.007; PFI: *p* < 0.001), LIHC (OS: *p* < 0.001; DSS: *p* = 0.011; DFI: *p* = 0.018; PFI: *p* = 0.006), MESO (OS: *p* < 0.001; DSS: *p* < 0.001; DFI: *p* = 0.036; PFI: *p* = 0.004), PCPG (PFI: *p* = 0.01), PRAD (DSS: *p* = 0.037; DFI: *p* < 0.001; PFI: *p* < 0.001), SARC (OS: *p* = 0.004; DSS: *p* = 0.002, DFI: *p* = 0.003; PFI: *p* = 0.013), UCEC (OS: *p* = 0.005; DSS: *p* < 0.001; DFI: *p* = 0.004; PFI: *p* < 0.001), and UVM (OS: *p* < 0.001; DSS: *p* < 0.001; PFI: *p* < 0.001) (Figures 2A, 3A, 4A, 5A).

The Kaplan-Meier cumulative curves displayed upregulated MRGBP expression was associated with poor prognosis in ACC (DFI: *p* = 0.041; PFI: *p* = 0.009), KIRC (OS: *p* < 0.001; DSS: *p* < 0.001; PFI: *p* < 0.001), KIRP (OS: *p* = 0.018; DSS: *p* = 0.002), LGG (OS: *p* < 0.001; DSS: *p* < 0.001;PFI: *p* < 0.001), LIHC (OS: *p* = 0.004; DSS: *p* = 0.037; DFI: *p* = 0.006; PFI = 0.006), LUAD (DFI: *p* = 0.006) MESO (OS: *p* < 0.001; DSS: *p* = 0.003; DFI: *p* = 0.019; PFI: *p* = 0.002), THCA (DSS: *p* = 0.04), PCPG (OS: *p* = 0.09; DSS: *p* = 0.027; PFI: *p* = 0.01), PRAD (DFI: *p* = 0.002; PFI: *p* < 0.001), SARC (DSS: *p* = 0.048, DFI: *p* = 0.041), UCEC (OS: *p* = 0.002; DSS: *p* < 0.001; DFI: *p* = 0.003; PFI: *p* < 0.001), and UVM (OS: *p* < 0.001; DSS: *p* < 0.001; PFI: *p* < 0.001) (Figures 2–5). However, high levels of MRGBP expression had longer survival time in patient with DLBC (OS: *p* = 0.016; DSS: *p* = 0.04) (Figures 2B,3B), while Cox regression analysis showed no statistical significance (Figures 2A, 3A).

Next we used Kaplan-Meier Plotter, which has data from GEO, EGA, and TCGA, to further analyze MRGBP-related survival including OS and RFS. The results shown in the Supplementary Figure 2 were mostly consistent with those obtained above, and MRGBP was a deleterious prognostic factor in BLCA (OS: HR = 1.66, logrank *P* = 0.0075), HNSC (OS: HR = 1.58, logrank *P* = 0.0015), KIRC (OS: HR = 2.13, logrank *P* < 0.001), KIRP (OS: HR = 2.46, logrank *P* = 0.0025; RFS: HR = 2.17, logrank *P* = 0.046), LIHC (OS: HR = 2.1, logrank *P* < 0.001;RFS: HR = 1.87, logrank *P* < 0.001), LUAD (OS: HR = 1.52, logrank *P* = 0.0071), SARC (OS: HR = 1.99, logrank *P* < 0.001; RFS: HR = 1.99, logrank *P* = 0.0062), UCEC (OS: HR = 2.21, logrank *P* < 0.001; RFS: HR = 2.33, logrank *P* = 0.0011). However, for ESCA, a high expression of MRGBP was found to be beneficial for overall survival (OS: HR = 0.07, logrank *P* < 0.001) but detrimental for relapse free survival (RFS: HR = 8.35, logrank *P* = 0.03).

### Association Between MORF4-Related Gene-Binding Protein Expression and Clinical Characteristics in Various Cancers

Next, we explored the association between the expression of mRNA and clinical feature in 33 cancers. Expression of MRGBP in different age groups showed significant differences in ESCA, KIRC, LGG, LIHC, LUAD, OV, SARC, STAD, and UCEC. Among them, less MRGBP expression was shown in patients over 65 years of age in ESCA, LIHC, and LUAD, and more expressed in other above cancers (Supplementary Figure 3). Moreover, MRGBP expression was significantly in different clinical stages in 10 cancers types including ACC, BRCA, COAD, ESCA, HNSC, KIRC, LIHC, LUSC, TGCT, and UVW (Supplementary Figure 4). It appears that MRGBP expression is higher in patients with stage III and IV than in patients with stage I and II. In addition, the expression of MRGBP in different molecular subtypes of COAD, GBM, HNSC, KIRP, LGG, LUSC, OV, PCPG, PRAD, READ, SKCM, STAD ACC, BRCA, and UCEC was significantly different (Supplementary Figure 5).

### MORF4-Related Gene-Binding Protein Copy Number Alterations and Mutations in Multiple Cancers

**FIGURE 6:**
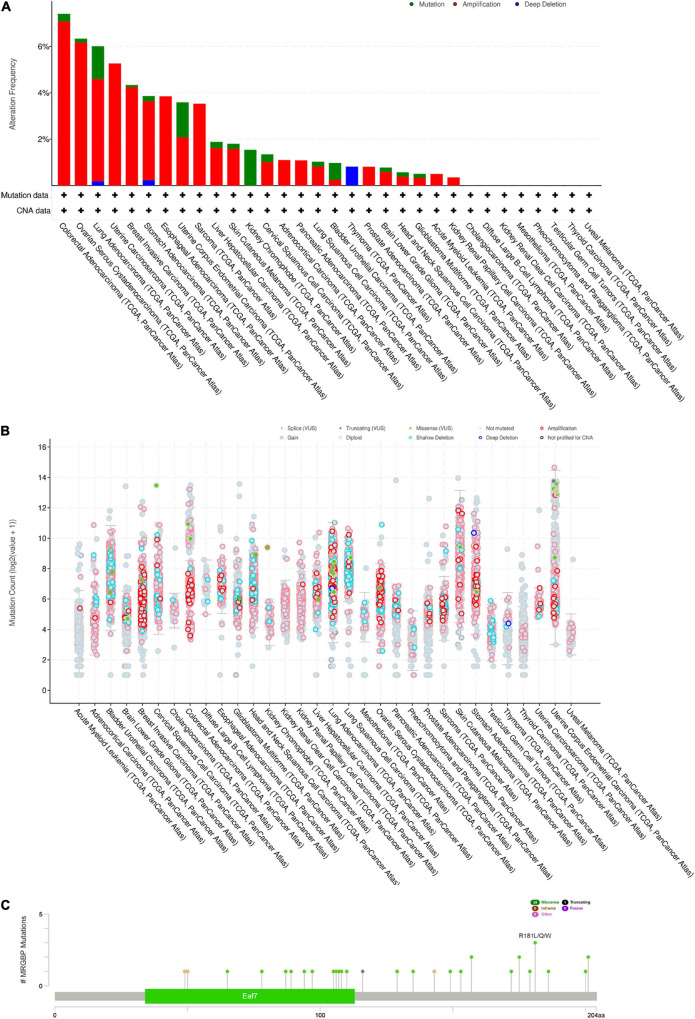
MRGBP Copy Number Alterations and Mutations. **(A)** MRGBP mutation level from the cBioPortal database. **(B)** MRGBP mutation frequency in multiple TCGA pan-cancer studies according to the cBioPortal database. **(C)** Mutation diagram of MRGBP in different cancer types across protein domains from the cBioPortal database.

We investigated the copy number alterations of MRGBP using the cBioPortal database. In all cancers, we found that the mutation frequency was relatively higher in colorectal adenocarcinoma and ovarian serous cystadenocarcinoma compared with other cancers. Amplification was the highest type of alteration, followed by mutation and deep deletion (Figures 6A,B). We identified 28 missense sites and 1 truncation site in MRGBP situated between amino acids 0 and 204 through the cBioPortal database (Figure 6C). Also, we obtained detailed and comprehensive mutational information on MRGBP in multiple tumors including missense mutations, synonymous mutations, shift deletions, and other mutation types using COSMIC (Supplementary Figure 6). Missense mutations were evident in most cancers such a large intestine cancer, lung cancer, and skin cancer and synergistic mutations were relatively uncommon. Other tumors with smaller sample sizes of mutations also showed different types of mutations. Among MRGBP coding chain mutations, C > T and G > A types were the most common, and other types of mutations were rarely found in different cancers.

### Correlation of MORF4-Related Gene-Binding Protein Expression With Tumor Mutation Burden, Tumor Microsatellite Instability, and Some Specific Genes in Various Cancers

**FIGURE 7:**
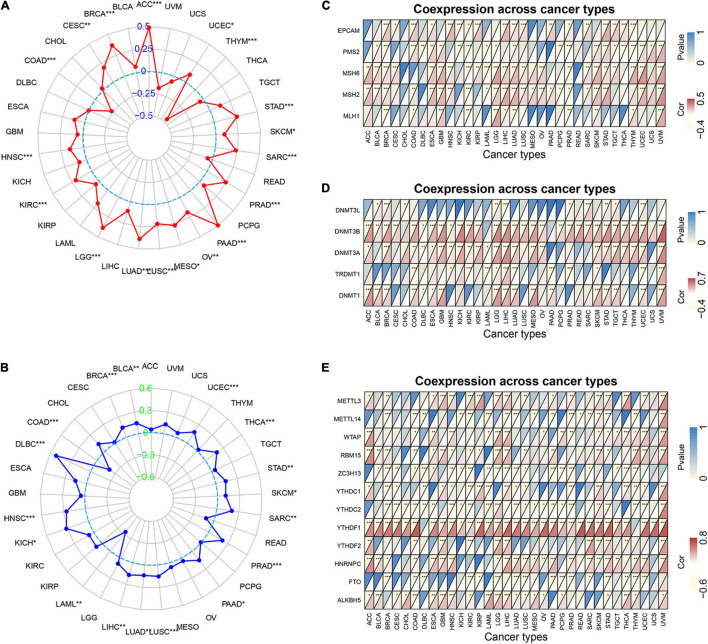
Relationship between MRGBP expression and tumor mutational burden **(A)**, microsatellite instability **(B)**, MMR genes **(C)**, DNA methyltransferases genes **(D)** and mRNA m6A related genes **(E)** in various tumors in TCGA database. The lower triangle in each tile indicates coefficients calculated by Pearson’s correlation test, and the upper triangle indicates log_10_-transformed *P*-value (***P < 0.001, **P < 0.01, *P < 0.05).

TMB is the number of somatic mutations in the tumor genome after removal of germline mutations, and high TMB shows better immunotherapeutic effects (Choucair et al., 2020). Defective DNA mismatch repair causes errors in tumor cell DNA replication leading to high MSI, which is associated with the effectiveness of immunotherapy in patients (van Velzen et al., 2020). As shown in Figure 7A, MRGBP expression significantly related to TMB in UCEC, STAD, SKCM, SARC, PRAD, PAAD, OV, MESO, LUSC, LUAD, LGG, KIRC, HNSC, THYM, COAD, CESC, BRCA, and ACC (*P* < 0.05) and most of them were positively correlated with TMB except THYM, COAD, CESC, BRCA, and ACC. We further verified whether the expression of MRGBP was associated with MSI in various cancers. In general, mRNA level was positively associated with MSI in 15 types of cancer including UCEC, THCA, STAD, SKCM, SARC, PRAD, PAAD, LUSC, LUAD, LIHC, KICH, HNSC, THYM, DLBC, BRCA, and BLCA. Only a few tumors such as LAML and COAD are negatively correlated with MRGBP expression (Figure 7B).

Mismatch repair (MMR) is one of the most important DNA repair processes and loss of function of DNA mismatch repair genes can cause the accumulation of mismatches during DNA replication, leading to tumorigenesis (Baretti and Le, 2018). Therefore, we explored the correlations between the expression of five MMR genes (EPCAM, PMS2, MSH6, MSH2, and MLH1) and MRGBP levels. Our results showed that the level of MMR gene was positively related to MRGBP expression in most tumors (Figure 7C). Methylation of DNA is an important epigenetic modification and abnormal methylation can lead to tumor development and somatic cell mutations. As shown in Figure 7D, in most tumors, MRGBP expression levels were positively associated with the expression of DNMT1, TRDMT1, DNMT3A, DNMT3B, and DNMT3L, especially DNMT3B. N6-methyladenosine (m6A), one of the most common RNA modifications, plays an important role in almost all important biological processes, including tumorigenesis and tumor progression. The modification level of m6A is dynamically regulated by a methyltransferase (writers), binding protein (readers), and demethylase (erasers). The results indicated that MRGBP expression was significantly related to the expression of these genes (Figure 7E).

### Association Between MORF4-Related Gene-Binding Protein Expression and Tumor Microenvironment in Human Cancers

**FIGURE 8:**
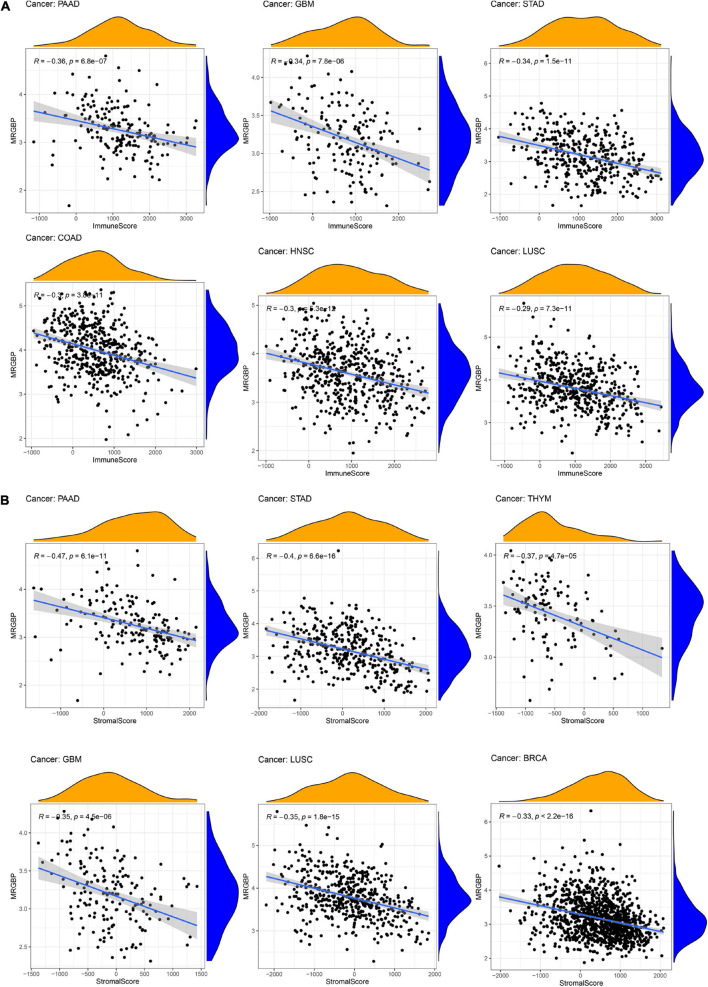
Association between MRGBP expression and immune scores **(A)** in PAAD, GBM, STAD, COAD, HNSC, and LUSC and stromal scores **(B)** in PAAD, STAD, THYM, GBM, LUSC, and BRCA.

Immune and stromal cells are the two major types of non-tumor components in the tumor microenvironment (TME). The degree of infiltration of them in TME has been reported to be of great value in the diagnosis of tumors and the response to immunotherapy (Wu and Dai, 2017). We then studied the relationship between the expression of MRGBP and TME in 33 cancers using the ESTIMATE algorithm. Our results indicated that MRGBP expression was significantly and negatively correlated with stromal scores and immune scores in COAD, GBM, HNSC, KIRP, LUAD, LUSC, PAAD, STAD, and UCEC. It suggested that the expression level of MRGBP was increased while the content of stromal or immune cells was decreased. We showed the six tumors with the highest correlation with stromal scores and immune scores (Figure 8) and the others are shown in Supplementary Figure 7.

### Association Between MORF4-Related Gene-Binding Protein Expression and Levels of Tumor Immune Cell Infiltration in Different Types of Cancer

**FIGURE 9:**
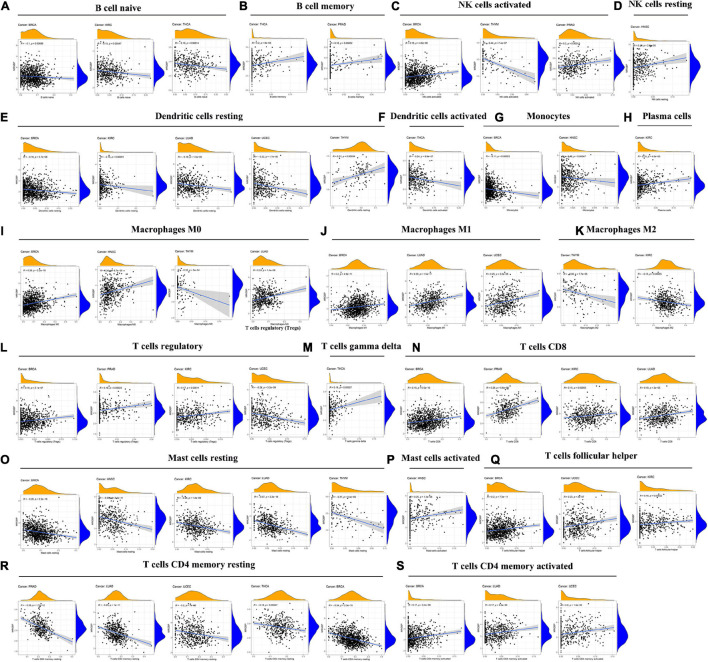
Association between MRGBP expression and the level of tumor infiltration of different immune cells in BRCA, HNSC, KIRC, LUAD, PRAD, THCA, THYM, and UCEC.

Subsequently, the correlation between MRGBP expression levels and the levels of 21 immune cell infiltrates in human cancers was studied. The results showed a significant correlation between the level of immune cell infiltration and MRGBP expression in almost all tumors. Among them, BRCA (*n* = 12), HNSC (*n* = 5), KIRC (*n* = 8), LUAD (*n* = 7), PRAD (*n* = 5), THCA (*n* = 5), THYM (*n* = 5), and UCEC (*n* = 6), which had the highest correlation coefficients between their immune cell infiltration levels and MRGBP levels, were used to further analyze (Figure 9).

Our results showed a negative correlation between MRGBP expression and the level of infiltrated naive B cells in KIRC, BRCA, and THCA but in terms of memory B cells, activated and resting NK cells, their infiltration levels were positively related to the MRGBP expression except for the infiltration of activated NK cells in THYM. For infiltrating resting and activated dendritic cells and monocytes, their levels were negatively correlated with MRGBP expression. Moreover, MRGBP expression levels appeared to be correlated with macrophage polarization. As shown in Figure 9, MRGBP expression was positively correlated with infiltrating M1 macrophages but negatively correlated with M2 macrophages. Also, there were different correlations between MRGBP expression levels and different infiltrating T cell subpopulations. The results showed a positive correlation between MRGBP expression and the levels of infiltrating activated memory CD4 + T cells, CD8 + T cells, follicular helper T cells, and regulatory T cells (except in UCEC); however, it was negatively related to the levels of resting memory CD4 T cells. MRGBP expression was negatively correlated with the levels of infiltrating resting mast cells in BRCA, HNSC, KIRC, LUAD, and THYM, but positively associated with infiltrating activated mast cells in HNSC. The relationship between MRGBP expression and infiltrating immune cells in other types of cancer is included in Supplementary Figure 8.

### Co-expression of Immune-Related Genes With MORF4-Related Gene-Binding Protein in Pan-Cancers

**FIGURE 10:**
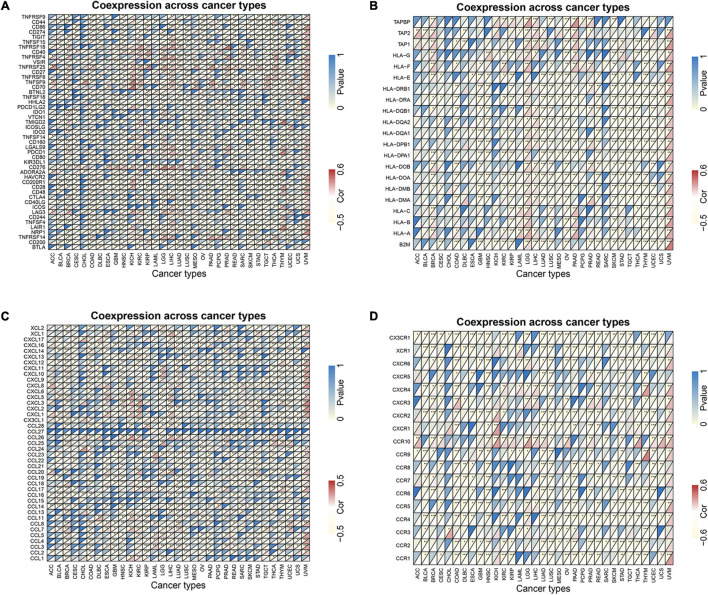
Co-expression of immune-related genes with MRGBP in pan-cancers. **(A)** Co-expression of immune checkpoint related genes with MRGBP. **(B)** Co-expression of MHC genes with MRGBP. **(C)** Co-expression of chemokine genes with MRGBP. **(D)** Co-expression of chemokine receptor genes with MRGBP.

Co-expression analyses were used to explore the relationship between MRGBP expression and immune-related genes including immune checkpoints, MHC, chemokine, and chemokine receptor proteins genes in 33 tumors. As is shown in Figure 10, we observed that most of the immune-related genes were associated with MRGBP expression and the majority were negatively related to the expression of MRGBP in different types of tumor except UVM. This suggests an interaction between MRGBP and immune checkpoints. Also, we investigated the expression difference between responders and non-responders according to TISIDB, which showed MRGBP expression was not significantly different between immunotherapy responders and non-responders (Supplementary Table 2).

### Enrichment Analysis of MORF4-Related Gene-Binding Protein-Related Partners

**FIGURE 11:**
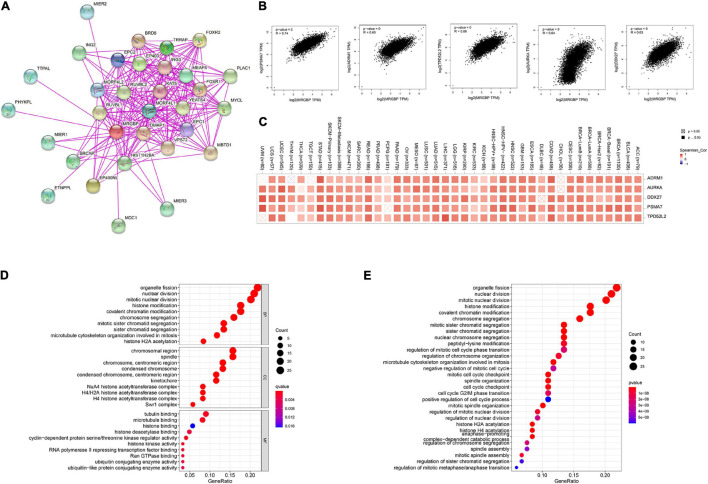
Enrichment analysis of MRGBP-related partners. **(A)** The PPI network of MRGBP is constructed by STRING database. **(B)** Correlation analysis between MRGBP and main interacting genes in TCGA using GEPIA2 tool. **(C)** Corresponding heatmap between the MRGBP expression and the main interacting genes in 33 cancer types. **(D)** The barplot and bubble of GO enrichment analysis. **(E)** The barplot and bubble of KEGG enrichment analysis. KEGG, Kyoto Encyclopedia of Genes and Genomes; GO, Gene Ontology; BP, biological process; CC, cellular component; MF, molecular function.

We screened MRGBP binding proteins and MRGBP expression-related genes for a series of pathway enrichment analyses to investigate the molecular mechanisms of MRGBP genes in tumorigenesis and tumor progression. As shown in the PPI network (Figure 11A), we obtained a total of 30 proteins that bind predominantly to MRGBP and these are supported by experimental evidence in STING. Also, we explored the correlation between MRGBP and the top 100 genes that related to MRGBP expression using the GEPIA2 tool, the results showed that MRGBP had the most significant correlation with PSMA7 (*R* = 0.74; *p* < 0.001), ADRM1 (*R* = 0.69; *p* < 0.001), TPD52L2 (*R* = 0.66; *p* < 0.001), AURKA (*R* = 0.64; *p* < 0.001) and DDX27 (*R* = 0.63; *p* < 0.001) (Figure 11B). Heatmap data also indicated a positive correlation between MRGBP and the above five genes in most cancer types (Figure 11C).

We next performed GO function annotation and KEGG pathway analysis using data from GSEA. The results of GO functional annotation showed MRGBP related genes were involved in the regulation of the cell cycle such as organelle fission, nuclear division, mitotic nuclear division, and histone modification. According to KEGG pathway enrichment analysis, MRGBP-related genes were positively correlated with organelle fission, nuclear division, mitotic nuclear division, histone modification which were consistent with the results of GO analysis (Figures 11D,E).

To better understand the potential mechanisms of MRGBP in cancer, we estimated the functional status of MRGBP in the CancerSEA database. As shown in Supplementary Figure 9, MRGBP has been studied at the single cell level in 13 types of tumors. We found that MRGBP was positively associated with DNA repair (cor = 0.479, *P* = 0.007), while negatively correlated with angiogenesis (cor = −0.363, *P* = 0.049) in acute lymphoblastic leukemia. MRGBP was negatively associated with invasion (cor = −0.433, *P* < 0.001) in ovarian cancer. MRGBP was positively associated with inflammation (cor = 0.329, *P* = 0.012) and quiescence (cor = 0.323, *P* = 0.013), while negatively correlated with DNA repair (cor = −0.406, *P* = 0.002) in colorectal cancer.

### Preliminary Verification of MORF4-Related Gene-Binding Protein Signature in Lower Grade Glioma and LIHC

**FIGURE 12:**
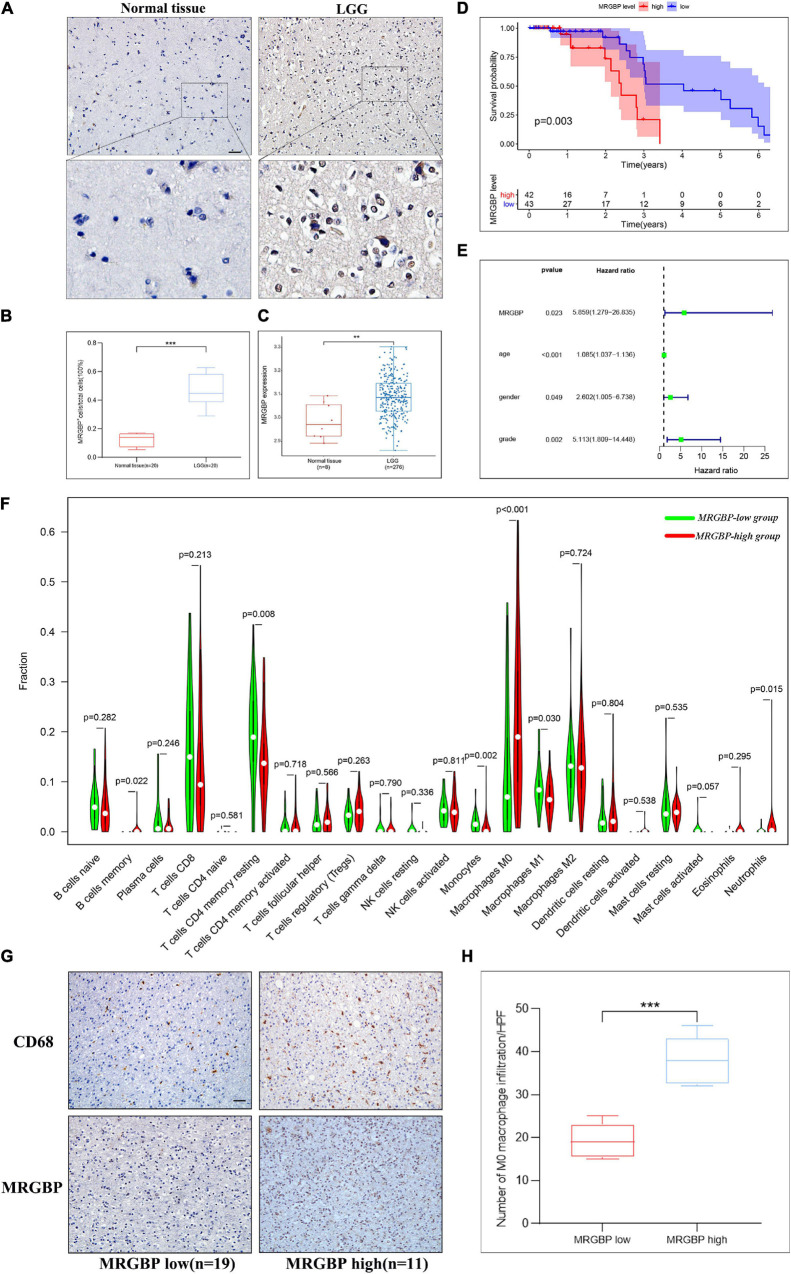
Preliminary verification of MRGBP signature in LGG. **(A)** Representative images of MRGBP immunohistochemistry in LGG. The picture below shows a 16× magnification. scale bars = 50 μm. **(B)** Quantification analysis showed higher MRGBP expression in LGG. **(C)** Validation of MRGBP expression by GSE16011. **(D)** Association between MRGBP expression and prognosis in GSE4412. **(E)** Univariate independent prognostic analysis in GSE4412. **(F)** Relative abundance of tumor-infiltrating immune cells in the MRGBP low expression group vs. MRGBP high expression group. **(G)** Representative images of CD68 immunohistochemistry in the MRGBP low expression group and MRGBP high expression group. scale bars = 50 μm. **(H)** Quantification analysis showed higher levels of M0 macrophage infiltration in the MRGBP high expression group (***P < 0.001, **P < 0.01).

Notably, in LGG and LIHC, high expression of MRGBP was significantly associated with poor prognosis including OS, DSS, DFI, and PFI (*p* < 0.001), suggesting that elevated MRGBP has significant predictive power for prognosis. Hence, we identified LGG and LIHC as representative cancer types for subsequent analysis. At first, we evaluated the difference in MRGBP expression in LGG and normal tissues using IHC. The results showed MRGBP was significantly upregulated (Figures 12A,B). We also verified the expression level of MRGBP in the GEO database (**GSE16011**) and the results were consistent with our findings (Figure 12C). Also, the results of IHC showed MRGBP was highly expressed in COAD and READ compared with normal tissues (Supplementary Figure 10A). Moreover, we investigated the prognostic value of MRGBP in LGG using the Chinese Glioma Genome Altas (CGGA) and GEO dataset (**GSE 4412**). The results of CGGA showed a high expression of MRGBP was related to a detrimental effect on overall survival (Supplementary Figure 10B). Similarly, increased expression of MRGBP predicted poor overall survival according to the GEO dataset (Figure 12D), which is generally consistent with our survival analysis of the TCGA database. Next, we conducted a univariate independent prognostic analysis using the GEO dataset, the expression level of MRGBP, age, gender, and grade were risk factors used to determine OS (Figure 12E) and it is consistent with the results from TCGA (Supplementary Figure 10C) except gender.

**FIGURE 13:**
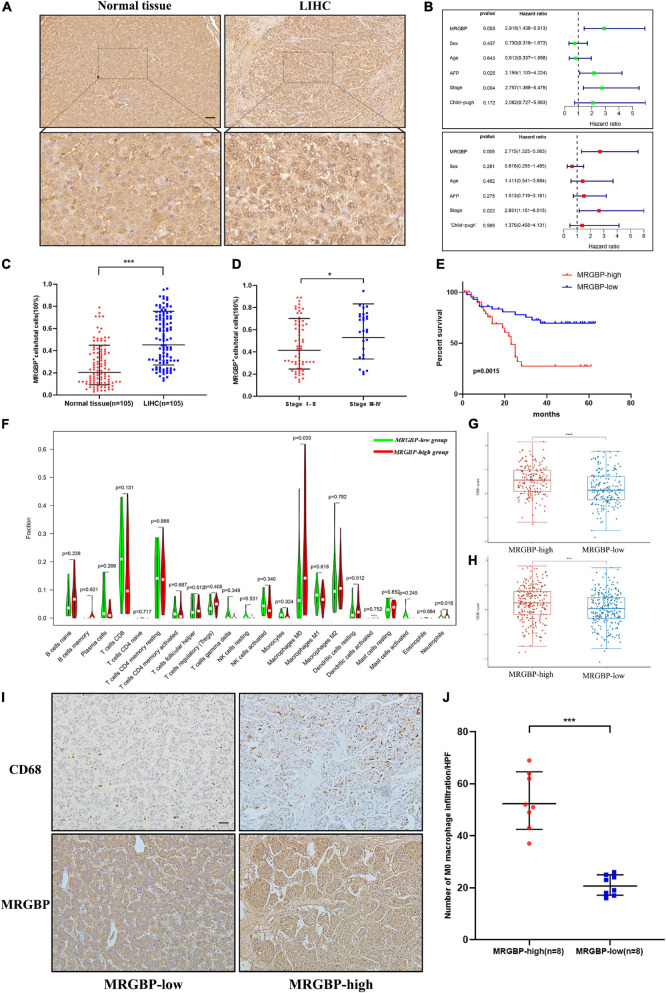
Preliminary verification of MRGBP signature in LIHC. **(A)** Representative images of MRGBP immunohistochemistry in LIHC. The picture below shows the enlarged images. scale bars = 50 μm. **(B)** Univariate and multivariate independent prognostic analysis of our LIHC data. **(C)** Quantification analysis showed higher MRGBP expression in LIHC. **(D)**. Quantification analysis showed higher MRGBP expression in stage III-IV patients. **(E)** Association between MRGBP expression and prognosis. **(F)** Relative abundance of tumor-infiltrating immune cells in the MRGBP low expression group vs. MRGBP high expression group. **(G,H)** TIDE scores in the high MRGBP and low MRGBP groups at LIHC and LGG. **(I)** Representative images of CD68 immunohistochemistry in the MRGBP low expression group and MRGBP high expression group. scale bars = 50 μm. **(J)** Quantification analysis showed higher levels of M0 macrophage infiltration in the MRGBP high expression group (****P < 0.0001, ***P < 0.001, *P < 0.05).

Next, we investigated the correlation between MRGBP and LIHC by detecting MRGBP protein expression in microarrays of liver tumor tissues containing 105 LIHC tissues and adjacent normal liver tissues by IHC staining. The results showed that MRGBP was significantly overexpressed in LIHC compared with adjacent normal tissues (*p* < 0.001) (Figures 13A,C and Supplementary Table 3). 90 patients containing clinical features and survival information were selected for further study of the association between MRGBP expression and clinical features and prognosis (Supplementary Table 3). We found that MRGBP expression was higher in stage III and IV patients compared to stage I and II (*p* = 0.04) (Figure 13D). In female, age ≥ 60, AFP ≥ 400 and Child-pugh B subgroups, MRGBP tended to be highly expressed although there was no statistically significant difference (*p* > 0.05) (Supplementary Figure 11). The survival curves revealed that patients with high expression of MRGBP had an unfavorable OS (*p* = 0.0015) (Figure 13E). Moreover, the results of univariate and multivariate COX analyses also identified MRGBP as a risk factor for OS (Figure 13B). The prognostic value of MRGBP in subgroups of LIHC patients with different clinical characteristics was further explored (Table 2). MRGBP correlated with poor prognosis for OS in male patients and patients with age < 60, AFP less than 400, earlier clinical stage and child-pugh A.

**TABLE 2:** Univariate survival analysis of MRGBP expression in subgroups with different clinical parameters.

Clinical parameters	n	Hazard ratio (95% CI)	*p*-value
**Sex**			
Male	75	3.688 (1.639–8.297)	**0.002**
Female	15	1.028 (0.213–4.969)	0.973
**Age**			
<60	70	3.806 (1.688–8.584)	**0.001**
≥60	20	0.908 (0.162–5.077)	0.912
**AFP**			
<400	48	4.586 (1.524–13.799)	**0.007**
≥400	42	1.959 (0.781–4.918)	0.152
**Stage**			
I–II	63	2.730 (1.129–6.602)	**0.026**
III–IV	27	2.518 (0.750–8.452)	0.135
**Child-pugh**			
A	82	2.893 (1.377–6.079)	**0.005**
B	8	1.674 (0.168–16.681)	0.660

*Bold values represent a statistical difference (p <0.05).*

Furthermore, we evaluated the abundance of TILs in the LGG and LIHC microenvironment using the TCGA gene expression data. The results showed that the levels of M0 macrophages were significantly higher in the MRGBP-high group in both LGG and LIHC, while the numbers of M1 macrophage, and resting memory CD4 T cells were significantly lower in the MRGBP-high group than MRGBP-low group in LGG. Lower levels of CD8 T cells infiltration were seen in patients in the MRGBP-high group of LGG and LIHC, although no significance was demonstrated (Figures 12F, 13F). Subsequently, TIDE algorithm (Jiang et al., 2018) was used to predict the potential ICB response. Our results showed elevated TIDE scores in MRGBP-high group in LIHC (Figure 13G) and LGG (Figure 13H), suggesting poor efficacy of immune checkpoint blockade therapy in the patients with high expression of MRGBP. The relationship between MRGBP expression and M0 macrophage infiltration level was next analyzed. We used serial paraffin sections from the same case to verify the relationship between CD68 and MRGBP expression levels. Representative images are shown in Figures 12G, 13I and it showed that in the group with high MRGBP expression levels, CD68 staining was stronger compared to the MRGBP-low group. MRGBP levels were positively correlated with the number of M0 macrophages (Figures 12H, 13J).

## Discussion

MRGBP, a component of the NuA4 histone acetyltransferase complex, is involved in the transcriptional activation of selected genes mainly through acetylation of nucleosomal histones H4 and H2A. MRGBP is upregulated in several types of cancers such as colorectal, cervical, prostate, pancreatic, and cutaneous squamous cell carcinomas and has been proved to increase replication, induce apoptosis, reduce growth and promote aggressiveness. There was also evidence that upregulation of MRGBP was associated with worse survival. All this evidence indicated that MRGBP was associated with tumor development as well as patient prognosis. However, no studies were exploring the role of MRGBP in a variety of cancers. In this report, we performed a multifaceted analysis of MRGBP in human cancers including its expression levels in tumors, normal tissues, and cell lines, the relationship between MRGBP expression and patient prognosis, TMB, and MSI. Also, we explored the correlation of MRGBP expression with TME, immune cell infiltration levels, and immune-related genes. Finally, we conducted a preliminary validation of our analysis results in the LGG.

Several studies have shown that MRGBP expression was increased in most cancer cell lines such as PDAC cell lines and the proliferative, migratory and invasive capacities of ASPC-1 and Mia PaCa-2 were significantly reduced (Ding et al., 2017). Another study showed that MRGBP expression was higher in most prostate cancer cell lines than in normal prostate cell lines and was able to enhance their aggressiveness (Ito et al., 2014). Similarly, elevated MRGBP expression was observed in colorectal cancer cell lines (Yamaguchi et al., 2011). Next, we analyzed the expression levels of MRGBP in a variety of normal as well as tumor tissues using multiple databases. The results showed that gene levels of MRGBP were significantly higher in 20 tumors compared to normal tissue. MRGBP expression in colorectal, prostate, and lung cancers is consistent with their previous studies (Yamaguchi et al., 2010; Ito et al., 2018; Dai et al., 2019) and it was also confirmed by our immunohistochemical results in LGG (Figure 12A). These findings suggest that MRGBP may play an important role in the development of the above tumors and it may be a promising diagnostic biomarker in tumors. Some studies analyzed the changed genes in intestinal and diffuse gastric cancer in the TCGA ribonucleic acid sequence data and found that MRGBP was one of the most changed genes (Tanabe et al., 2020). Our results also showed that MRGBP expression was significantly different among different molecular subtypes in most types of tumors.

Also, we analyzed the relationship between the MRGBP expression and prognostic value in 33 types of cancer using TCGA databases and Kaplan-Meier plotter. Based on both Cox and KM survival analysis of TCGA, high MRGBP expression served as a detrimental prognostic factor in some types of cancer including DLBC, KIRC, KIRP, LGG, LIHC, MESO, PCPG, UCEC, UVM. Similarly, in pancreatic cancer, elevated expression of MRGBP was previously reported as positively associated with poor prognosis (Ding et al., 2017). Also, analysis of altered genes in 37 colorectal adenomas and 31 adenocarcinomas showed that MRGBP expression was significantly higher in carcinomas compared to adenomas (Carvalho et al., 2009). This revealed MRGBP played an important role in the progression of adenoma to cancer and it might have potential clinical applications as a highly specific biomarker for colorectal cancer. According to the Kaplan-Meier plotter, we found that elevated MRGBP expression was related to worse prognosis in all tumors with statistically different, especially in KIRC, KIRP, LIHC, and UCEC which is consistent with the previous results analyzed with TCGA. We also performed a preliminary validation in LGG using the GEO and CGGA database. The results were consistent with previous studies in TCGA. The results of our LIHC data analysis also indicated that patients with high MRGBP expression were associated with shorter survival times and that MRGBP expression levels were an independent prognostic factor. These results suggested that MRGBP could be used as a potential prognostic biomarker for multiple tumors, especially for LGG and LIHC.

Furthermore, MRGBP expression in relation to age was observed in certain types of cancers. MRGBP was lowly expressed in older patients (age > 65) with ECSC, LIHC, and LUAD, while in KIRC, LGG, OV, SARC, STAD, and UCEC, lower MRGBP expression was associated with young patients. These results have implications for the selection of immunotherapy regimens for patients in different age groups. In most cancers, MRGBP expression was associated with tumor stage and was significantly higher in patients with advanced stages. MRGBP expression was significantly higher in stage III and IV tumors. Our tissue microarray results confirmed a higher number of MRGBP-positive cells in stage III and IV patients.

TMB is a newly discovered quantifiable clinical metric that holds promise for predicting tumor response to immunotherapy (Samstein et al., 2019). High TMB has been reported to predict the efficacy of tumor immunotherapy in patients with lung cancer (Devarakonda et al., 2018), melanoma (Alexandrov et al., 2013), and colorectal cancers (Lee et al., 2019). Patients with high TMB had significantly longer overall survival compared to patients with low TMB after immune checkpoint blockade treatment (Samstein et al., 2019). MMR, an important DNA repair mechanism, plays an important role in maintaining genomic stability. Mutations or deletions in MMR genes lead to defects in gene function and microsatellite instability (MSI) (Boland and Goel, 2010). Lee et al. (2016) reported that the PD-1 monoclonal antibody pembrolizumab improved survival in patients with high MSI cancers compared to those with low MSI cancers. Similarly, colorectal cancer patients with high MSI gain better benefit from targeted monoclonal antibody nivolumab therapy (Overman et al., 2017). Our results showed that MRGBP expression was positively correlated with TMB and MSI in most cancer types. Patients with high MRGBP expression in these tumors may respond well to immunotherapy.

DNA and RNA methylation (m6A) are two important nucleic acid modifications that play an important role in the regulation of gene expression and participate in many biological processes (Szigeti et al., 2018). Both of them can affect tumor proliferation, differentiation, tumorigenesis, invasion, and metastasis by regulating proto-oncogenes and oncogenes. In our research, MRGBP expression was significantly associated with DNA methyltransferase genes and M6A RNA methylation-related genes, particularly DNMT3B and YTHFD1. It has been revealed that lysosomal protease mRNA could be bound by YTHDF1 to inhibit its cross-presentation ability to antigens in dendritic cells. This suggested that YTHDF1 may be a possible therapeutic target for immunotherapy (Han et al., 2019). Overall, MRGBP may influence tumor development and ultimately patient prognosis by regulating DNA methyltransferase gene and M6A RNA methylation. Also, it may be a potential target to predict the efficacy of immunotherapy in patients.

Tumor cells, which usually colonize normal tissues, can form a tumor microenvironment (TME) together with stromal cells, immune cells and their secretory factors and extracellular matrix (ECM) components. Studies have shown that immune cells and the cytokines produced by them, including immunosuppressive and inflammatory cytokines, play a dual role in promoting or preventing cancer development (Junttila and de Sauvage, 2013; Lianyuan et al., 2018; Wang et al., 2020). Nevertheless, few studies have been conducted on the role of MRGBP in the immune microenvironment. In our research, the results showed that MRGBP expression negatively correlated with stromal and immune cell content in COAD, GBM, HNSC, KIRP, LUAD, LUSC, PAAD, STAD, and UCEC according to ESTIMATE scores. Our research further clarifies that MRGBP expression was significantly related to the levels of multiple immune-related cells including NK cells, dendritic cells, M0 macrophages, M1 macrophages, M1 macrophages, regulatory T cells, CD4^+^ T cells, and CD8^+^ T cells. Among them, CD8^+^ T lymphocyte plays a tumor-killing function, while regulatory T cells attenuate effector T cell activity and promote immunosuppression in TME. M1-type macrophages secrete Th1 cytokines, which play a pro-inflammatory and anti-tumor role, while the M2 macrophages in TME promote angiogenesis and tumor invasion by secreting Th2 cytokines. Also, natural killer cells are capable of killing tumor-associated immune cells by releasing granzyme and perforin or by mediating antibody-dependent cytotoxicity with their Fc segment receptors. Dendritic cells are also affected by the hypoxic and inflammatory environment in the TME, which impairs their antigen-presenting activity (Gajewski et al., 2013; Cully, 2018; da Silva et al., 2019). We selected LGG and LIHC to further verify the relationship between MRGBP expression and the level of immune cell infiltration, and the results showed that in the high MRGBP expression group, the level of M0 macrophages was significantly higher, while M1 macrophages, CD4^+^ T and CD8^+^ T cells were lower compared to the low MRGBP group. These suggested that MRGBP regulated macrophage infiltration and might have a potential regulatory role in macrophage polarization. Our IHC analysis results confirmed that the high MRGBP expression group had more infiltration of macrophages. Besides, MRGBP expression was considered to be significantly associated with genes encoding immune checkpoints, MHC, chemokines, and chemokine receptor proteins in our study. The results of the TIDE algorithm showed that patients with low MRGBP expression have lower TIDE scores at LGG and LIHC, implying that patients with low MRGBP expression are more likely to benefit from immunotherapy. These results suggested that MRGBP may affect patient prognosis by modulating immune infiltration of tumors, and it may be a potential target for immunotherapy.

Finally, we performed a series of enrichment analyses by screening out genes closely related to MRGBP expression, and the results showed that MRGBP contributed to tumor development by regulating ganelle fission, nuclear division, mitotic nuclear division, and histone modifications. Similarly, previous studies have found that MRGBP was associated with DNA replication, microchromosome maintenance, and cell division in colorectal cancer (Yamaguchi et al., 2011).

In summary, our results demonstrated high MRGBP expression in a variety of tumors and revealed the correlation between MRGBP expression and tumor progression. Moreover, MRGBP expression was significantly correlated with poor prognosis, TMB, MSI, and immune cell infiltration in most types of cancer. Besides, MRGBP expression level was an independent prognostic factor for LGG and LIHC. Low MRGBP expression was associated with lower TIDE scores, which suggested a better immunotherapy efficacy. These results revealed that MRGBP can serve as a potential prognostic biomarker. It plays an important role in tumor immune infiltration in various tumors and may be a potential target for immunotherapy, especially in LGG and LIHC.

## Data Availability Statement

The original contributions presented in the study are included in the article/Supplementary Material, further inquiries can be directed to the corresponding author/s.

## Ethics Statement

The studies involving human participants were reviewed and approved by the Ethics Committee of the Renmin Hospital of Wuhan University Ethics Committee of Tongji Hospital, Tongji Medical College, Huazhong University of Science and Technology. The patients/participants provided their written informed consent to participate in this study.

## Author Contributions

DC, LZ, and YG came up with the design and conception, prepared the material, conducted the experiments, collected the data, and analyzed the data. DC wrote the first draft of the manuscript. All authors commented on previous versions of the manuscript, read, and approved the final manuscript.

## Conflict of Interest

The authors declare that the research was conducted in the absence of any commercial or financial relationships that could be construed as a potential conflict of interest.

## Publisher’s Note

All claims expressed in this article are solely those of the authors and do not necessarily represent those of their affiliated organizations, or those of the publisher, the editors and the reviewers. Any product that may be evaluated in this article, or claim that may be made by its manufacturer, is not guaranteed or endorsed by the publisher.

## Supplementary Material

Supplementary Figure 1 mRNA expression of MRGBP gene in normal human tissues and tumor cell lines. **(A)** Normal tissue expression. **(B)** mRNA expression in 38 kinds of tumor cell lines from the CCLE database.

Supplementary Figure 2 Kaplan-Meier survival curves comparing the high and low expression of MRGBP gene in various cancer types in Kaplan-Meier Plotter.

Supplementary Figure 3 Relationship between MRGBP expression and age in ESCA, KIRC, LGG, LIHC, LUAD, OV, SARC, STAD, and UCEC.

Supplementary Figure 4 Relationship between MRGBP expression and tumor stage in ACC, BRCA, COAD, ESCA, HNSC, KIRC, LIHC, LUSC, TGCT, and UVW.

Supplementary Figure 5 mRNA expression of MRGBP in different molecular subtypes of COAD, GBM, HNSC, KIRP, LGG, LUSC, OV, PCPG, PRAD, READ, SKCM, STAD ACC, BRCA, and UCEC.

Supplementary Figure 6 Pie chart showing the percentage of the different mutation types of MRGBP in various cancers according to the COSMIC database.

Supplementary Figure 7 Association between MRGBP expression and immune scores and stromal scores in multiple cancers.

Supplementary Figure 8 Correlation of MRGBP expression and infiltrating immune cells in multiple cancers.

Supplementary Figure 9 Functional state of MRGBP across 13 types of cancer. The red plots indicated that MRGBP was positively correlated and the blue plots indicated that MRGBP was negatively correlated with the functional state identified by CancerSEA.

Supplementary Figure 10 **(A)** Representative images of MRGBP immunohistochemistry in COAD and READ. **(B)** Association between MRGBP expression and prognosis in CGGA datasets. **(C,D)** Hazard ratio and *P*-value of constituents involved in univariate and multivariate Cox regression and some parameters of the MRGBP genes.

Supplementary Figure 11 **(A)** Immunohistochemical staining of MRGBP on LIHC tissue microarrays. **(B)** Expression of MRGBP in subgroups with different clinical parameters.

Supplementary Table 1 Database and its website.

Supplementary Table 2 The expression of MRGBP between immunotherapy responders and non-responders.

Supplementary Table 3 Expression levels of MRGBP and the clinical information contained in LIHC tissue microarrays.
