# Supplementary figures and images for: Prognostic Value and Immunological Role of MORF4-Related Gene-Binding Protein in Human Cancers
Source: Front Cell Dev Biol. 2021 Sep 29;9:703415. doi: 10.3389/fcell.2021.703415 (PMC8511499; doi:10.3389/fcell.2021.703415)

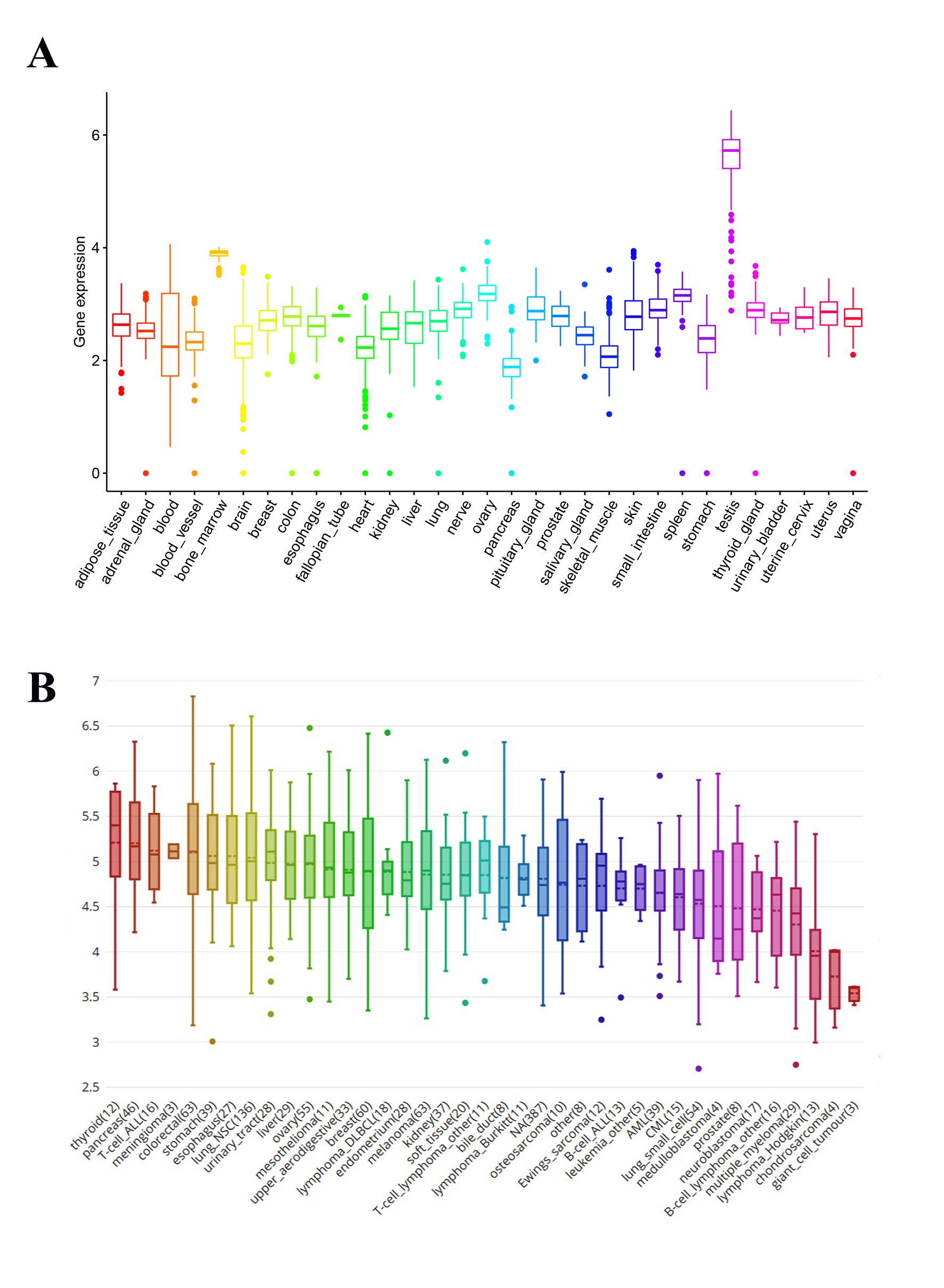

Supplement: Supplementary Figure 1 — mRNA expression of MRGBP gene in normal human tissues and tumor cell lines. (A) Normal tissue expression. (B) mRNA expression in 38 kinds of tumor cell lines from the CCLE database. [file Image_1.TIF]

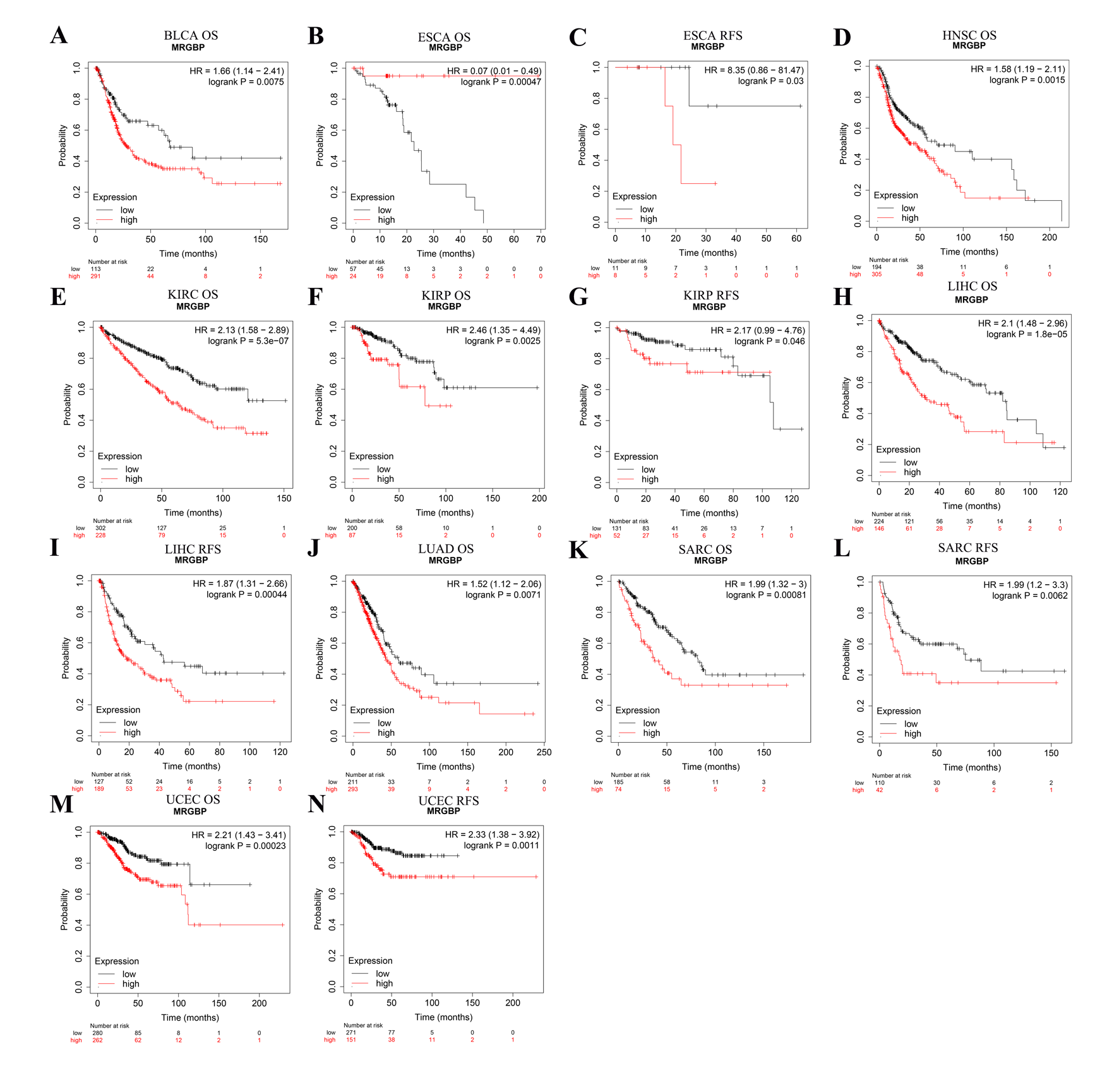

Supplement: Supplementary Figure 2 — Kaplan-Meier survival curves comparing the high and low expression of MRGBP gene in various cancer types in Kaplan-Meier Plotter. [file Image_2.TIF]

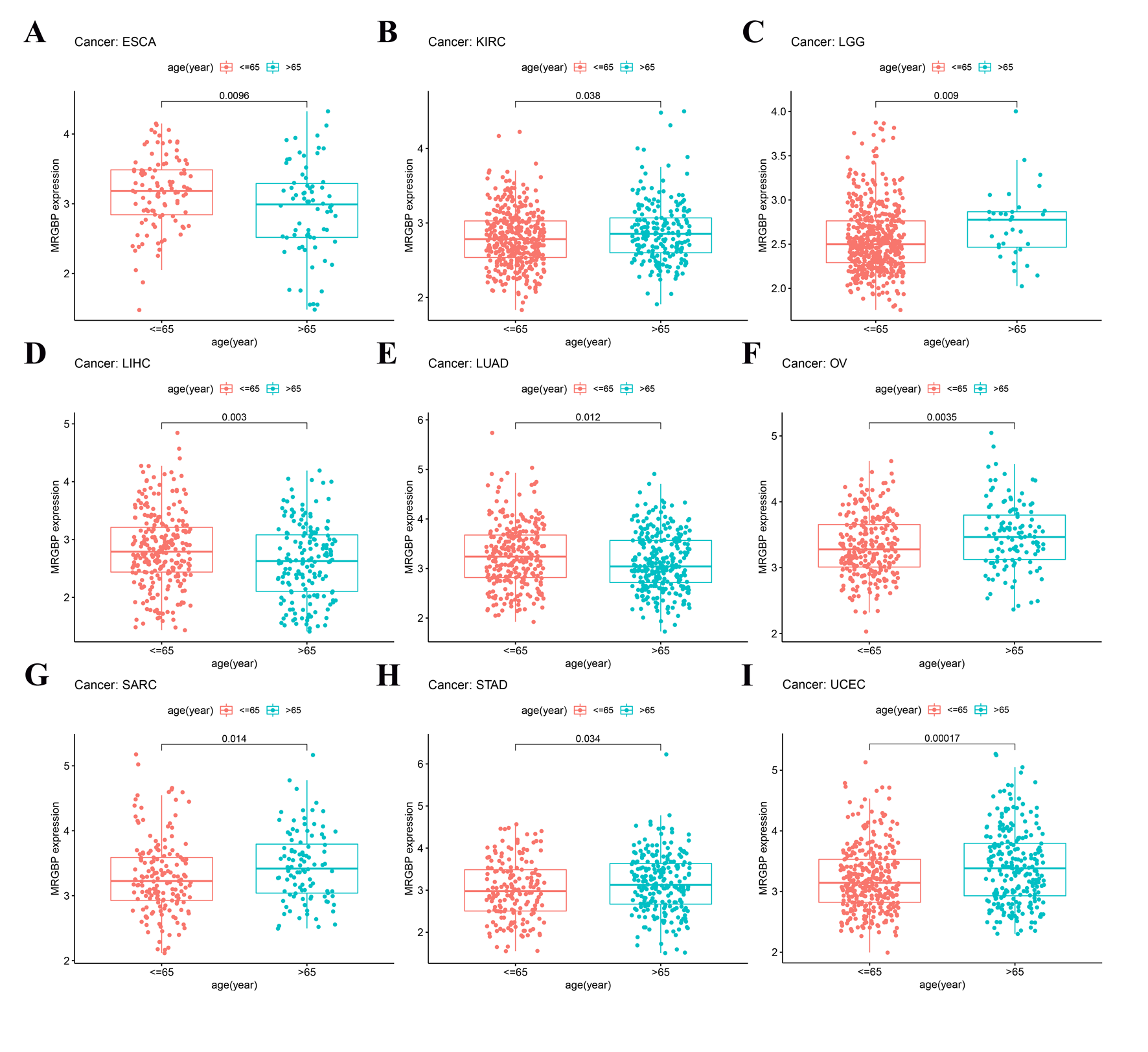

Supplement: Supplementary Figure 3 — Relationship between MRGBP expression and age in ESCA, KIRC, LGG, LIHC, LUAD, OV, SARC, STAD, and UCEC. [file Image_3.TIF]

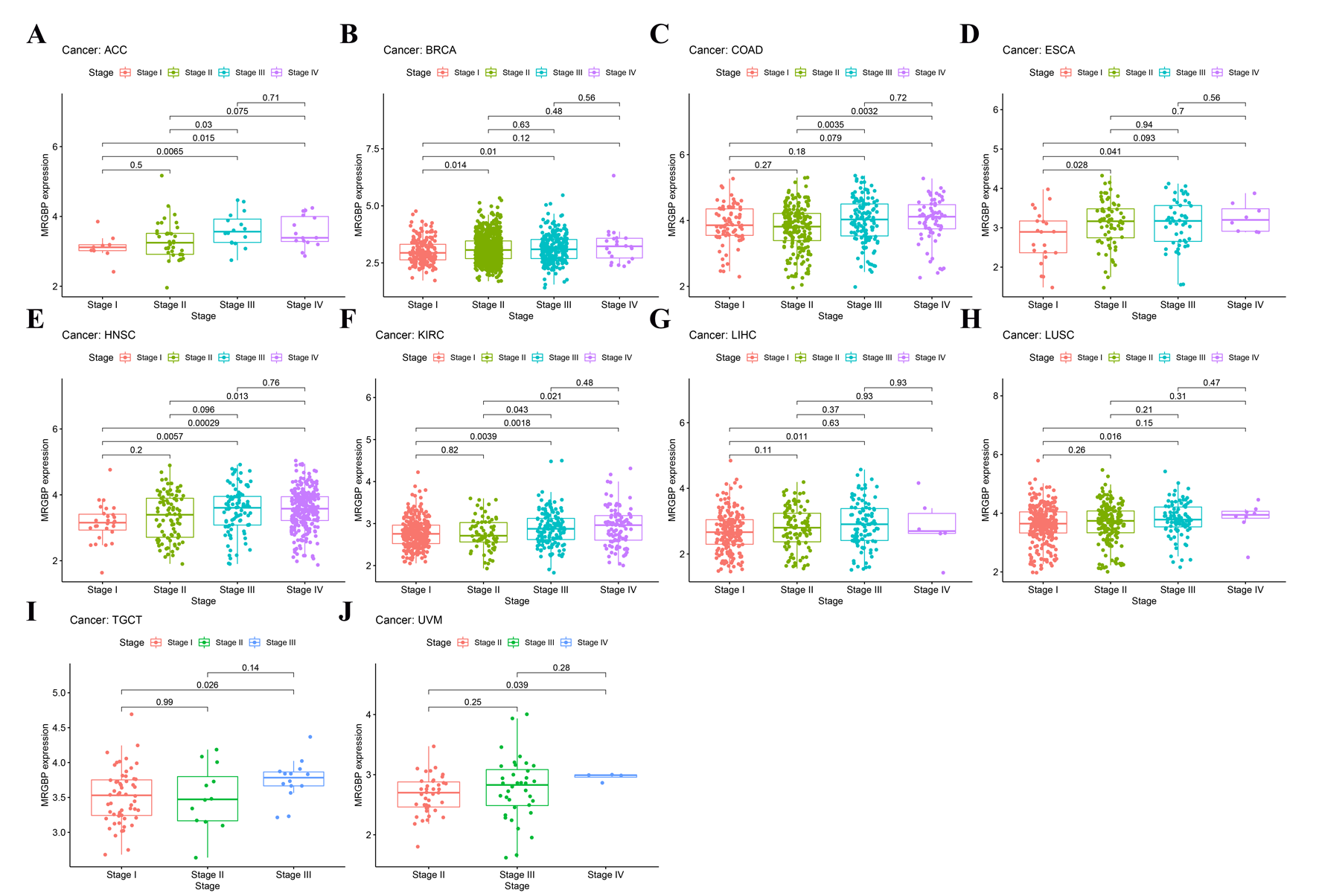

Supplement: Supplementary Figure 4 — Relationship between MRGBP expression and tumor stage in ACC, BRCA, COAD, ESCA, HNSC, KIRC, LIHC, LUSC, TGCT, and UVW. [file Image_4.TIF]

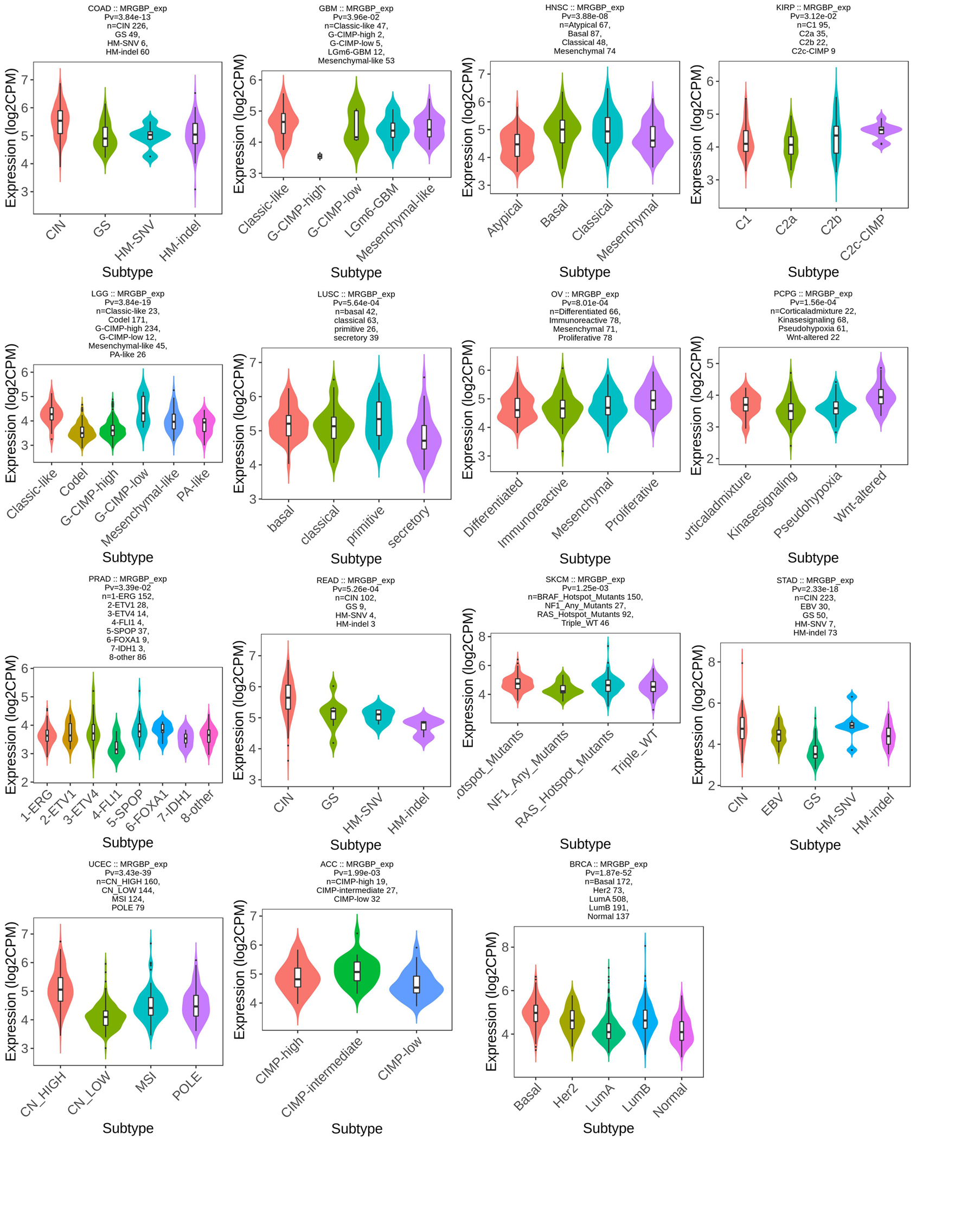

Supplement: Supplementary Figure 5 — mRNA expression of MRGBP in different molecular subtypes of COAD, GBM, HNSC, KIRP, LGG, LUSC, OV, PCPG, PRAD, READ, SKCM, STAD ACC, BRCA, and UCEC. [file Image_5.TIF]

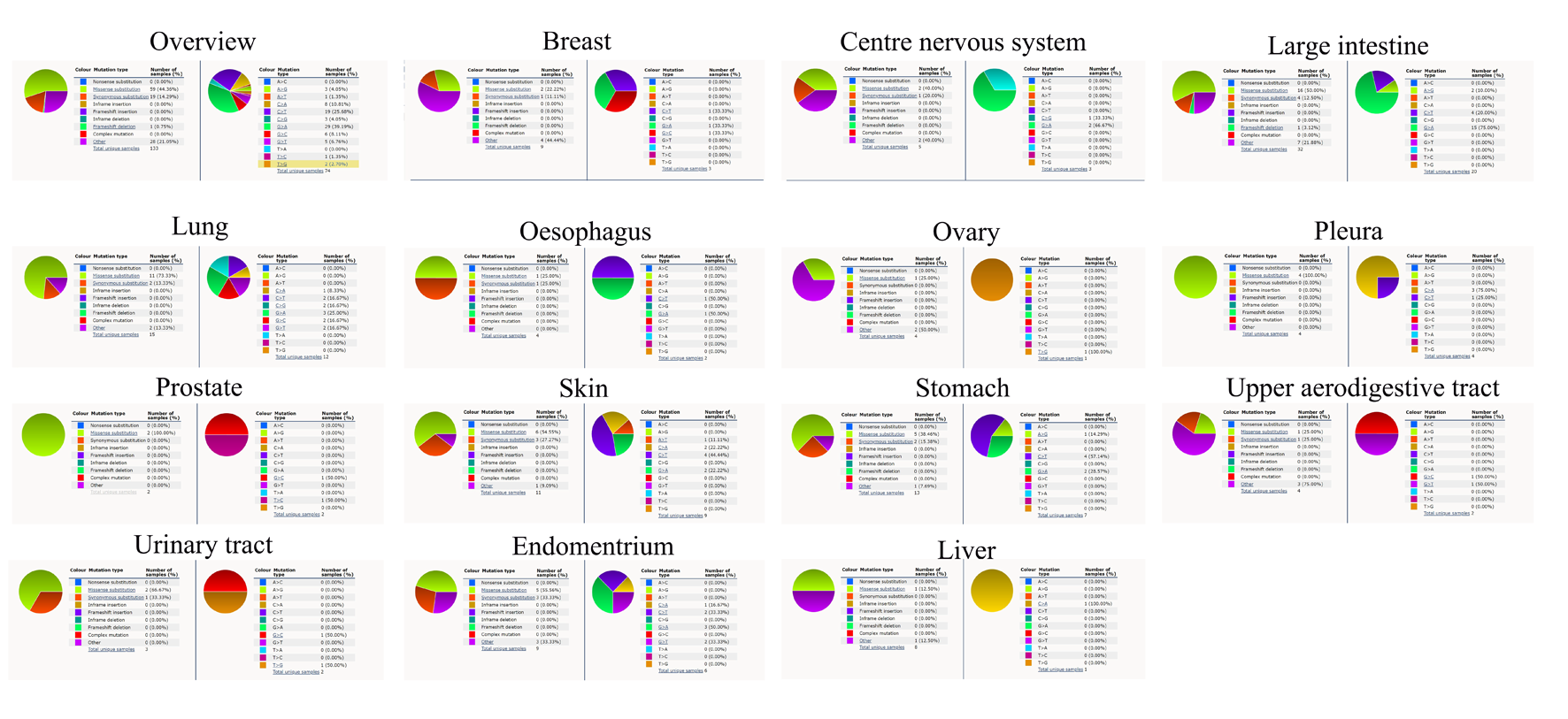

Supplement: Supplementary Figure 6 — Pie chart showing the percentage of the different mutation types of MRGBP in various cancers according to the COSMIC database. [file Image_6.TIF]

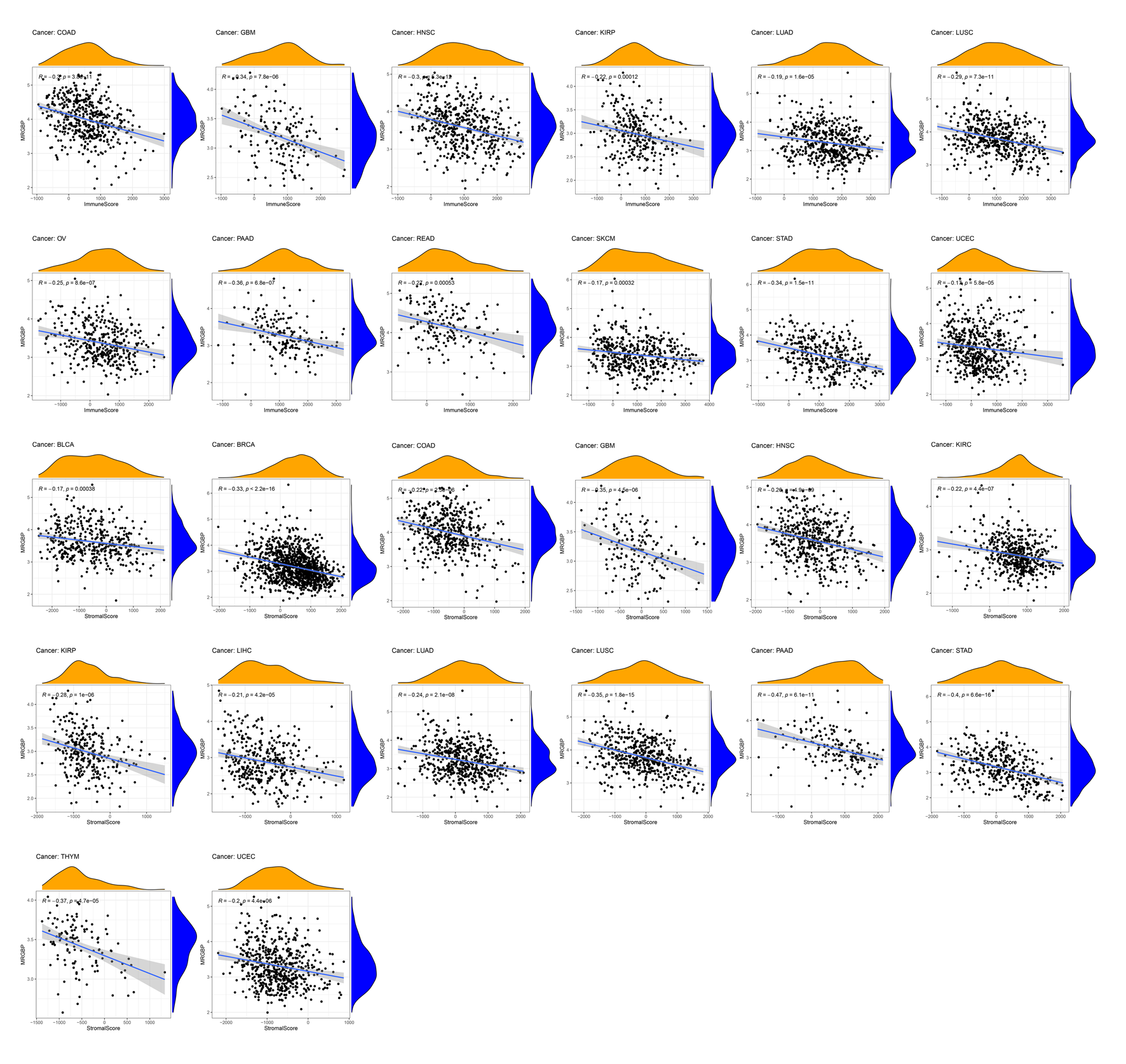

Supplement: Supplementary Figure 7 — Association between MRGBP expression and immune scores and stromal scores in multiple cancers. [file Image_7.TIF]

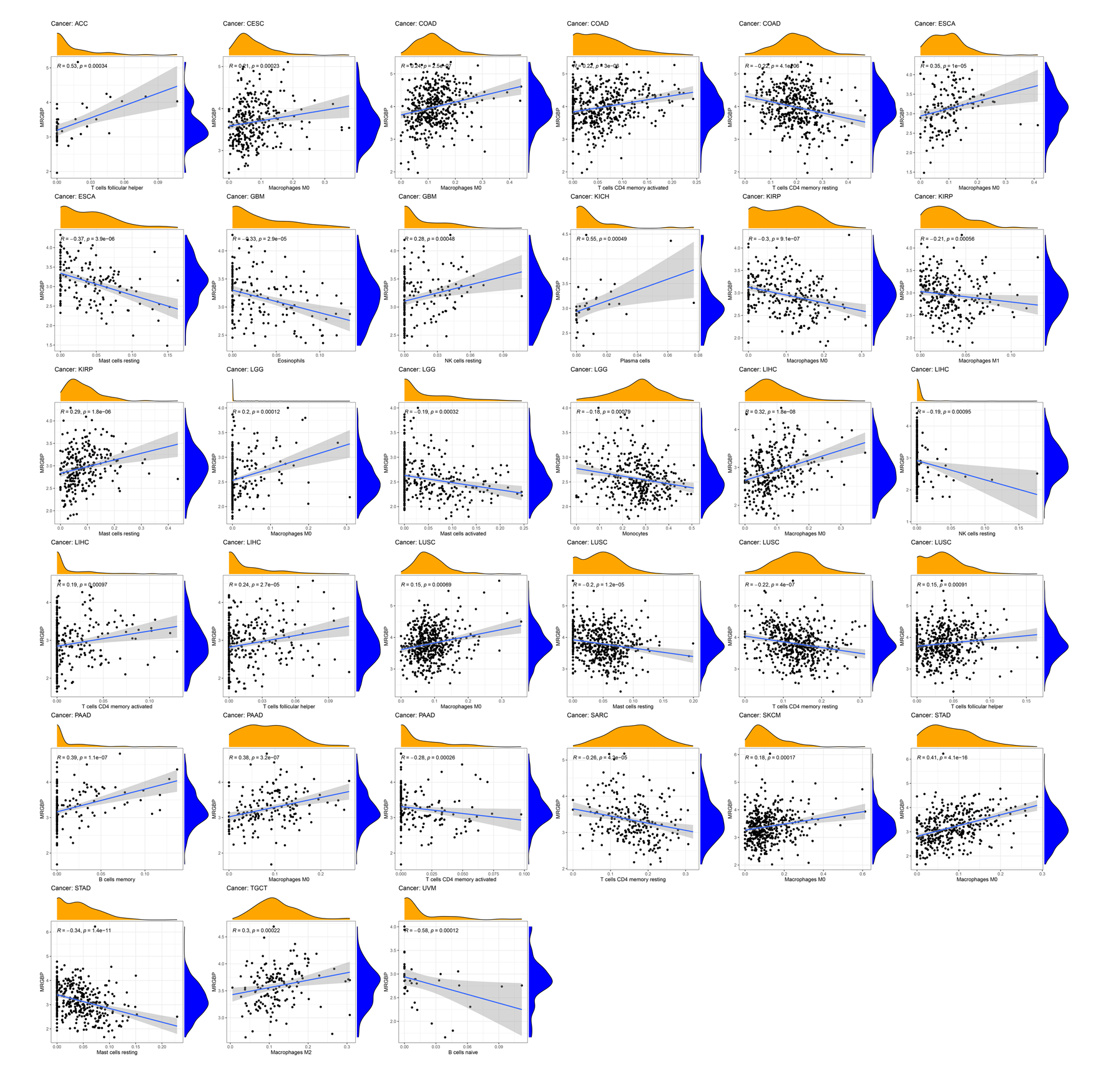

Supplement: Supplementary Figure 8 — Correlation of MRGBP expression and infiltrating immune cells in multiple cancers. [file Image_8.TIF]

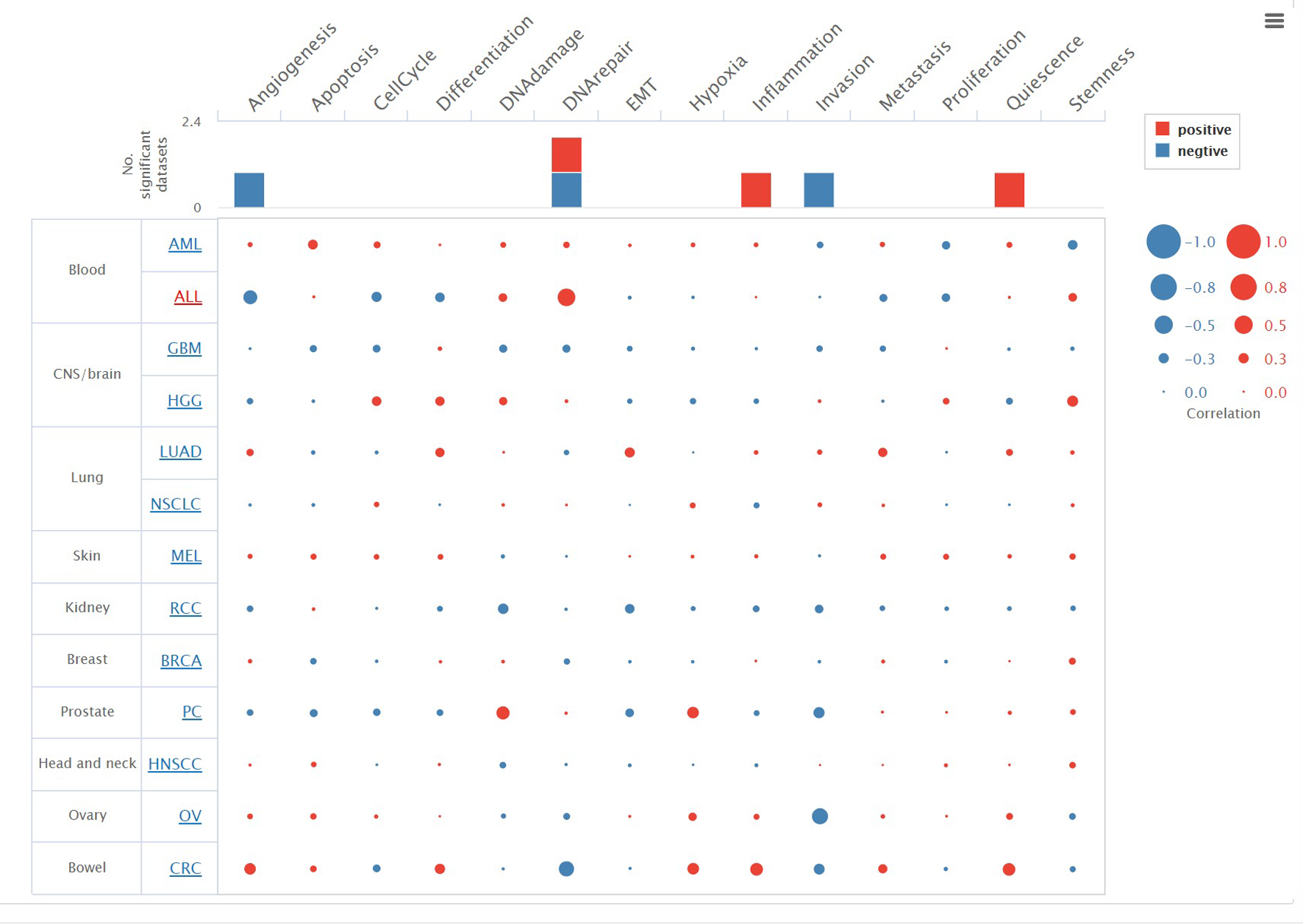

Supplement: Supplementary Figure 9 — Functional state of MRGBP across 13 types of cancer. The red plots indicated that MRGBP was positively correlated and the blue plots indicated that MRGBP was negatively correlated with the functional state identified by CancerSEA. [file Image_9.TIF]

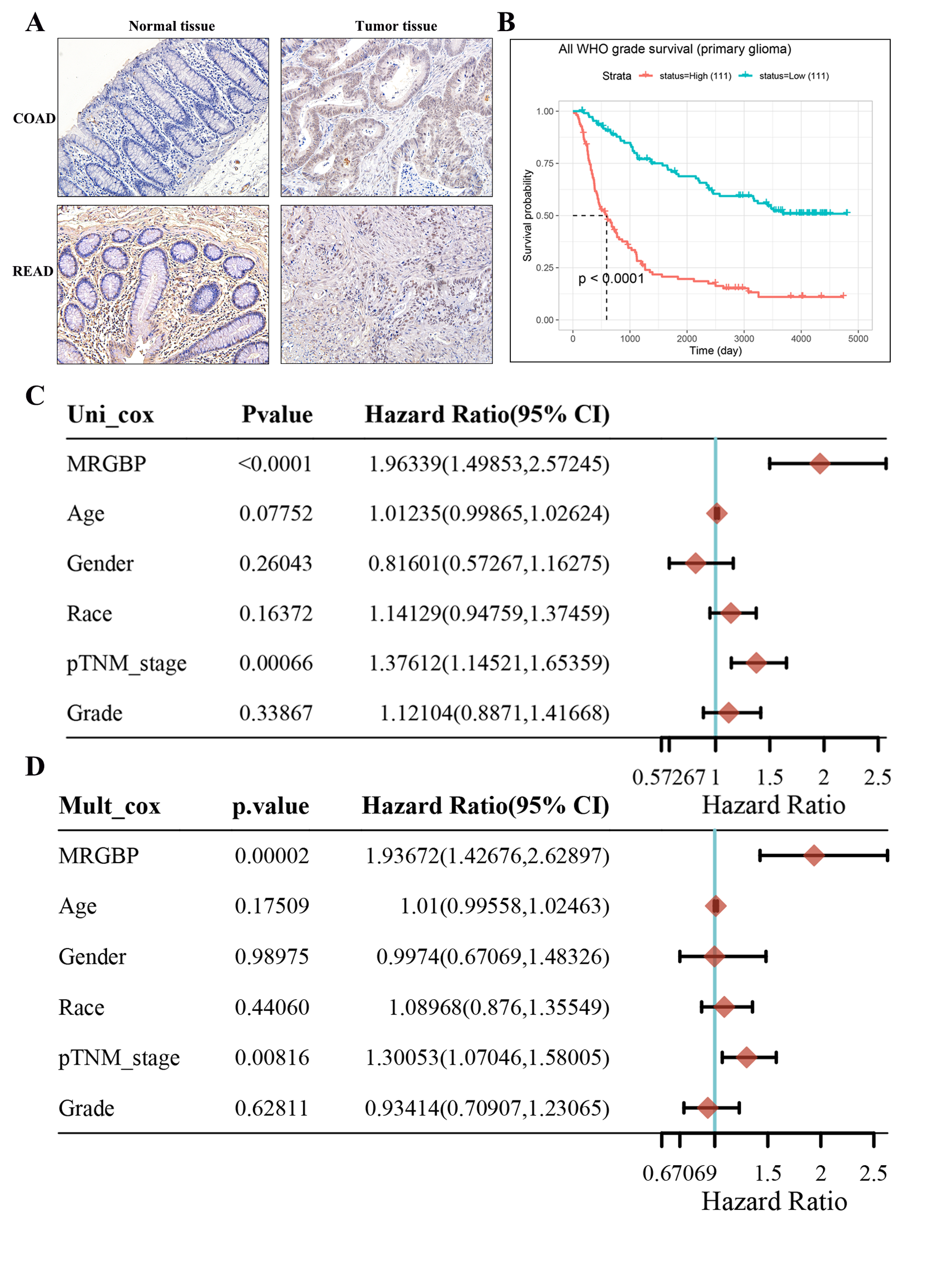

Supplement: Supplementary Figure 10 — (A) Representative images of MRGBP immunohistochemistry in COAD and READ. (B) Association between MRGBP expression and prognosis in CGGA datasets. (C,D) Hazard ratio and P-value of constituents involved in univariate and multivariate Cox regression and some parameters of the MRGBP genes. [file Image_10.TIF]

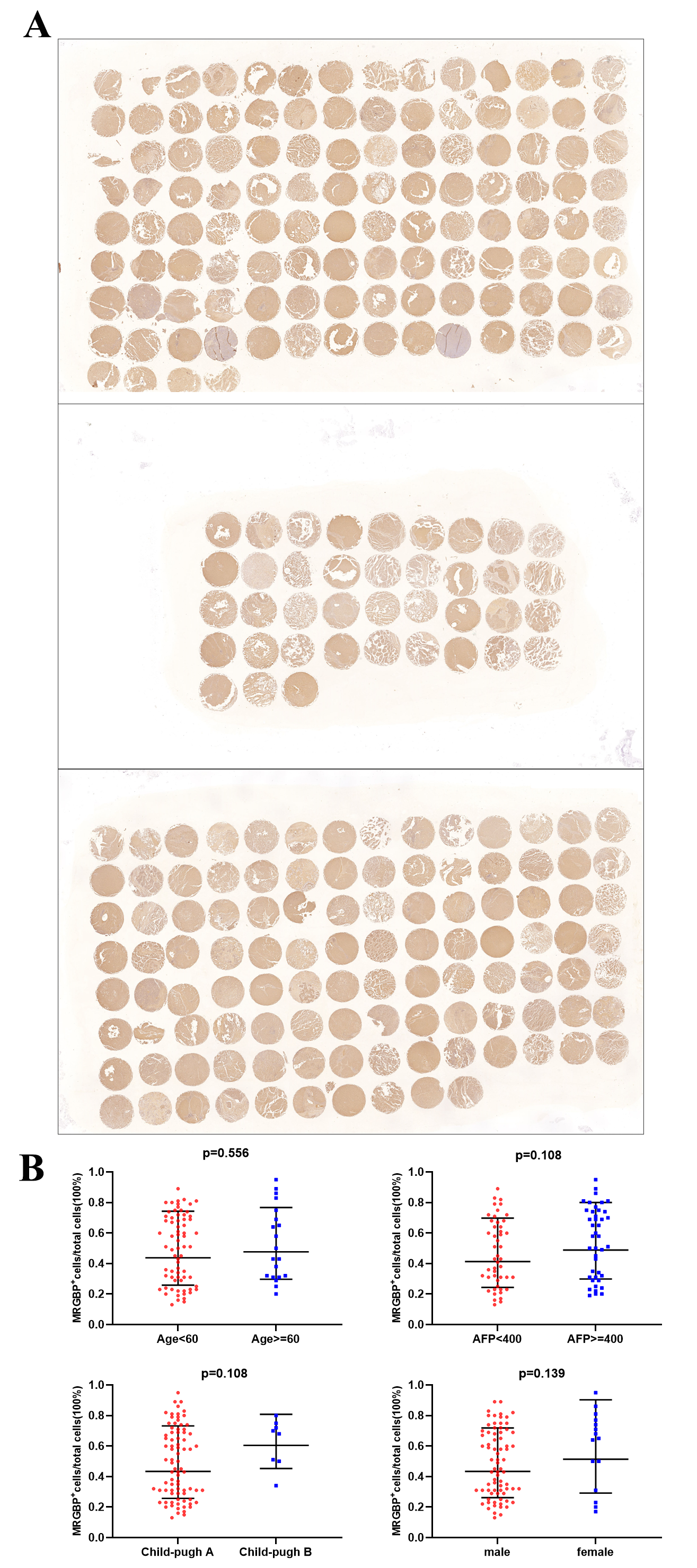

Supplement: Supplementary Figure 11 — (A) Immunohistochemical staining of MRGBP on LIHC tissue microarrays. (B) Expression of MRGBP in subgroups with different clinical parameters. [file Image_11.TIF]
