# Supplementary material for: Prognostic Value and Immunological Role of MORF4-Related Gene-Binding Protein in Human Cancers
Source: Front Cell Dev Biol. 2021 Sep 29;9:703415. doi: 10.3389/fcell.2021.703415 (PMC8511499; doi:10.3389/fcell.2021.703415)
Supplement: Supplementary Table 1 — Database and its website. [file Table_1.docx]

| Table S1. Database and its website |  |
| --- | --- |
| Databases | Website |
| Xena Browser | https://xenabrowser.net/datapages/ |
| The Cancer Cell Line Encyclopedia | https://portals.broadinstitute.org/ccle/about |
| ONCOMINE | https://www.oncomine.org/resource/main.html |
| TISIDB | http://cis.hku.hk/TISIDB/ |
| cBioPortal | http://www.cbioportal.org/ |
| Catalogue of Somatic Mutations in Cancer | https://cancer.sanger.ac.uk/cosmic/ |
| DNA Methylation Interactive Visualization Database | http://119.3.41.228/dnmivd/ |
| Gene Expression Omnibus | https://www.ncbi.nlm.nih.gov/geo/ |
| Kaplan-Meier Plotter | https://kmplot.com/analysis/ |
| CellMiner | https://discover.nci.nih.gov/cellminer/ |
| GEPIA2 | http://gepia2.cancer-pku.cn/#index |
| STRING | https://www.string-db.org/ |
| Gene Set Enrichment Analysis | https://www.gsea-msigdb.org/gsea/downloads.jsp |
| Sangerbox | http://sangerbox.com/ |
| The Cancer Genome Atlas Program  TIMER2 | https://www.cancer.gov/about-nci/organization/ccg/research/structural-genomics/tcga  http://timer. cistrome.org/ |
